# Multivariate Bayesian meta-analysis: joint modelling of multiple cancer types using summary statistics

**DOI:** 10.1186/s12942-020-00234-0

**Published:** 2020-10-17

**Authors:** Farzana Jahan, Earl W. Duncan, Susana M. Cramb, Peter D. Baade, Kerrie L. Mengersen

**Affiliations:** 1 ARC Centre of Excellence in Mathematical and Statistical Frontiers, School of Mathematical Sciences, Science and Engineering Faculty, Queensland University of Technology, Brisbane, QLD 4001 Australia; 2 Institute of Health and Biomedical Innovation, Queensland University of Technology, Brisbane, QLD 4001 Australia; 3 Cancer Council Queensland, 553 Gregory Terrace, Fortitude Valley, Brisbane, QLD 4006 Australia

**Keywords:** Cancer incidence, Cancer atlas, Online estimates

## Abstract

**Background:**

Cancer atlases often provide estimates of cancer incidence, mortality or survival across small areas of a region or country. A recent example of a cancer atlas is the Australian cancer atlas (ACA), that provides interactive maps to visualise spatially smoothed estimates of cancer incidence and survival for 20 different cancer types over 2148 small areas across Australia.

**Methods:**

The present study proposes a multivariate Bayesian meta-analysis model, which can model multiple cancers jointly using summary measures without requiring access to the unit record data. This new approach is illustrated by modelling the publicly available spatially smoothed standardised incidence ratios for multiple cancers in the ACA divided into three groups: common, rare/less common and smoking-related. The multivariate Bayesian meta-analysis models are fitted to each group in order to explore any possible association between the cancers in three remoteness regions: major cities, regional and remote areas across Australia. The correlation between the pairs of cancers included in each multivariate model for a group was examined by computing the posterior correlation matrix for each cancer group in each region. The posterior correlation matrices in different remoteness regions were compared using Jennrich’s test of equality of correlation matrices (Jennrich in J Am Stat Assoc. 1970;65(330):904–12. 10.1080/01621459.1970.10481133).

**Results:**

Substantive correlation was observed among some cancer types. There was evidence that the magnitude of this correlation varied according to remoteness of a region. For example, there has been significant negative correlation between prostate and lung cancer in major cities, but zero correlation found in regional and remote areas for the same pair of cancer types. High risk areas for specific combinations of cancer types were identified and visualised from the proposed model.

**Conclusions:**

Publicly available spatially smoothed disease estimates can be used to explore additional research questions by modelling multiple cancer types jointly. These proposed multivariate meta-analysis models could be useful when unit record data are unavailable because of privacy and confidentiality requirements.

## Background

Cancer atlases are the geographical representation of cancer incidence, mortality or survival to describe the cancer burden scenario across/between areas of a country, sub-region or group of countries with accompanying descriptive and analytical statistics [1, 2]. The atlases are useful tools for showing geographic patterns of cancers [3] and have made significant contributions in cancer research [4]. A cancer atlas can be one of the methods to identify cancer patterns or risk factors [2]. Examples of early cancer atlases include, National Atlas of Disease Mortality in the United Kingdom [5], the Atlas of cancer mortality for U.S. counties, 1950–1969 [6], Atlas of U.S. cancer mortality among whites, 1950–1980 [7], U.S. cancer mortality rates and trends, 1950–1979 [8], Atlas of cancer mortality in the People’s Republic of China [9], Atlas of U.S. cancer mortality among non-whites, 1950–1980 [10] and; Atlas of cancer mortality in the European Economic Community [11]. Cancer atlases started to be published online in recent times, such as: Atlas of Cancer in India [12], NCI Cancer Atlas [13], Cancer Atlas of the United Kingdom and Ireland [14], the U.S. Atlas of Cancer Mortality [15], Atlas of Cancer in Queensland [16] and the Australian Cancer Atlas (ACA) [17]. These atlases not only provide important information about the geographical variation in cancer burden but can also motivate different etiological questions about cancers. Most of the available cancer atlases modelled each cancer separately (univariate modelling) to obtain age standardised rates or indirect standardised ratios for incidence and hazard ratios or similar for survival for each cancer across the small areas.

One recent cancer atlas is the ACA [17]. The ACA provides point and interval estimates of cancer incidence and relative survival for 20 cancers over 2148 small areas (Statistical area level 2, SA2 [18]) across Australia along with interactive maps to visualise geographic patterns in cancer incidence and survival. The estimates used to produce the maps are based on an underlying Bayesian spatial model of the observed population data aggregated to the SA2 level; for details of the underlying methodology, please see [19]. All the smoothed estimates of cancer incidence and survival available in the ACA were obtained by univariate modelling of each cancer type separately. To calculate the summary estimates for 14 cancer types (oesophageal, stomach, liver, pancreatic, cervical, uterine, ovarian, kidney, brain, thyroid, non-Hodgkin lymphoma, leukaemia, myeloma and head and neck), data on a 10-yr time period (2005–2014) were used. For the remaining six cancer types (bowel, lung, melanoma, breast, prostrate, all cancers combined), data on a 5-yr time period (2010–2014) were used.

There has been growing interest in joint modelling of two or more cancer types in order to explore the shared and divergent trends among the cancers in terms of geographic patterns and risk factors [20]. The most popular joint model for identifying the common risk factors of multiple disease is the shared component model [21], where instead of a multivariate model for jointly modelling two diseases, the underlying risk surface is separated into a disease specific risk component and a shared component. For example, Mahaki et al. [22] applied multivariate disease mapping of seven prevalent cancer types in Iran using a shared component model. A joint-analysis of the spatio-temporal variation of the six age-gender (three ages groups (0–14, 15–64, and 65 and over) and gender (male, female)) mortality risks was performed by [23] using a shared component spatio-temporal model. Bayesian shared component spatio-temporal models for male and female lung cancer was applied to analyse the spatio-temporal variation of lung cancer diagnosis [24, 25].

Other multivariate approaches for modelling multiple cancers are also available. Use of mixture factor models in modelling multivariate cancer outcomes was introduced by [26]. Hewson and Bailey [27] also developed a latent mixture model for modelling four types of carcinoma and explored the spatial correlation structures among the cancer types between 300 geographic units in England, Scotland and Wales. A spatio-temporal mixture model was proposed to analyse the space-time variation in respiratory cancers in the state of South Carolina [28]. Mezzetti M. [29] proposed a hierarchical Bayesian factor model for spatially correlated data to explain across and within county correlations of cancer incidence rates by assuming that all different cancer types (12 for females and 10 for males) share one or more spatially correlated common factors. The model was to age-standardised cancer incidence rates by sex in 56 counties of Scotland. Most of these modelling approaches used unit level data from population based cancer registries, but this data can be difficult to access due to confidentiality and privacy requirements of data custodians.

More recent work has proposed ways to use summary measures, instead of raw unit record files, when modelling, such as by applying an extended Gamma-Poisson model [30]. The authors showed an algorithm to extract data from several sources and analyse the summary statistics. However, the algorithm and model is applicable for univariate response variable. Additionally, Beranger and Sisson [31] proposed new statistical models for analysis of summary estimates for symbolic data analysis. These models considered any symbols, such as random lists, histogram or intervals, derived from aggregating individual level data and performed statistical inferences for the symbols. One of the limitations of the symbolic data analysis approach is the problem of evaluating high dimensional integral over data space. There is further scope for improvement to existing methods and development of new methods in order to model the estimated summary information without accessing the unit record data.

In an earlier study, Bayesian hierarchical meta-analysis models for each of the 20 cancers were fitted separately and the pattern of incidence according to remoteness categories (major cities, regional and remote areas) was explored [32]. The univariate meta-analysis model, if extended to accommodate multiple selected cancers in the same model, can be employed to identify possible association between selected cancers and could also help in detecting small areas where multiple cancer types have higher incidence rates jointly.

There has been only one study, to the best of our knowledge, which has studied the relationship between two cancers, namely colorectal and breast cancer, using summary measures from a cancer atlas to explore the factors responsible for the observed association [33]. This was a simplistic graphical comparison of ranked age-standardised cancer death rates, supplemented with a literature review to provide some etiologic hypotheses and suggest new opportunities of research in order to explore the association between the two cancers.

In the present study, instead of considering only two specific cancer types, multiple cancers from the ACA were chosen and the relationship is evaluated using posterior correlation matrices obtained by fitting a multivariate Bayesian hierarchical meta-analysis model. In addition to investigating the relationship among multiple cancers, the areas with higher risk of multiple cancers are also identified. The meta-analysis uses the spatially smoothed estimates from ACA, since these are publicly available. The proposed multivariate models in this study are expected to provide a more comprehensive understanding of relationships between the incidence of different cancer types. Using the hierarchical structure, we examine differences or similarities in observed relationships among groups of cancers across broad remoteness regions in Australia.

## Methods

The proposed multivariate Bayesian meta-analysis model is described in the context of the ACA. The ACA is a freely accessible and interactive online platform, showing the spatial variation in standardised incidence and survival for 20 cancer types across Australia (for a complete list, please see Appendix A1). The ACA provides the point estimates for the standardised incidence ratios (SIRs) and excess hazard ratios and their $95\%$ credible intervals for each of the 20 cancer types in each of 2148 geographical areas (SA2) covering Australia.

Whereas a typical meta-analysis combines outcomes from different studies, the proposed method adopts the same meta-analysis principles and techniques to combine the estimated summary measures from each of the 2148 areas. These summary measures, comprising estimated SIRs and corresponding 95% credible intervals, are results of Bayesian spatial models using observed cancer incidence data in each area. Hence, instead of modelling outputs from multiple studies, we are modelling outputs from multiple small areas.

### Model formulation

Let $y_{ijk}$ and $s^2_{ijk}$ denote the estimated mean and variance of the *log*(*SIR*) respectively for the $i{th}$ cancer, $j{th}$ small area and $k{th}$ category, where $i= 1,2,3,...,n$ and *n* is the number of cancers included in the multivariate model, $j=1,2,3,...,J$, *J* is the total number of areas and $k=1,2, ... , K$ and *K* is the number of categories of interest. In our analysis of the ACA, $J=2148$ and $K=3$ (the number of remoteness categories), where $k=1$ if the $j{th}$ SA2 is a major city, $k=2$ if the $j{th}$ SA2 is a regional area and $k=3$ if the $j{th}$ SA2 is a remote area, and *n* takes on different values according to the analysis; see below.

The remoteness information is obtained from the remoteness structure provided by Australian Bureau of Statistics in each Statistical Area level 1 (SA1, which aggregate to form SA2s) as a five-category index (major cities, inner regional, outer regional, remote and very remote) [34]. We assigned one remoteness area to each SA2 based on SA1 population sizes before combining the inner and outer regional areas, as well as remote and very remote areas, into regional and remote, respectively. Among the 2148 SA2s considered in the ACA, 1242 are major cities, 810 are regional and 96 are classified as remote areas.

In the ACA, the values of $y_{ijk}$ and $s_{ijk}^2$ are the outputs of a Bayesian spatial model. Hence, we model $y_{ijk}$ as follows:

1 $$\begin{aligned} y_{ijk} \sim N(\mu _{ijk},\sigma ^2_{ijk}) \end{aligned}$$

where, $\mu _{ijk}$ is the true value of the *log*(*SIR*) for the $i{th}$ cancer, $j{th}$ SA2 and $k{th}$ region with associated variance $\sigma ^2_{ijk}$. Here we are not modelling the raw data but the estimated statistics for each small area which are provided by the ACA.

Now, $\mu _{ijk}$ can be further modelled as a multivariate normal distribution:

2 $$\begin{aligned} \mu _{ij(k)} \sim MVN(\mu _{i(k)},\Sigma _{(k)}) \end{aligned}$$

where, $\mu _{i(k)}$ is the region-specific means for $k{th}$ region and $i{th}$ cancer and $\Sigma _{(k)}$ denotes the covariance matrix accounting for the covariance among the means in the same region and different cancers. This hierarchy is added in the model to address the research question involving identifying patterns of cancer incidence in different regions.

The region-specific means for the $i{th}$ cancer, $\mu _{i(k)}$ can be further modelled hierarchically (see Appendix A2), but for the sake of this study, we will consider modelling up to this level and will focus on the posterior means and the posterior covariance matrices associated with different cancers in each region. The aim is to explain how the relationship between the cancers varies with respect to major cities, regional and rural/remote areas.

The priors for the model parameters can be specified as follows:

$\sigma _{ijk}^2$ can take a prior that utilises the uncertainty information from the estimates available in the atlas as,

3 $$\begin{aligned} \sigma _{ijk}^2 \sim \frac{\nu s_{ijk}^2}{\chi _{(\nu )}^2} \end{aligned}$$

where $\chi _{(\nu )}^2$ denotes the $\chi ^2$ distribution with $\nu$ degrees of freedom. The degrees of freedom, $\nu$, of the $\chi ^2$ distribution are chosen to reflect the prior degree of certainty in these estimates [35]. Following the rationale of [35], a common choice of $\nu$ is 2, which will be used in this study.

The prior for the variance covariance matrix $\Sigma _k$ is described by an inverse Wishart distribution as

4 $$\begin{aligned} \Sigma _{(k)} \sim IW(V,n) \end{aligned}$$

where *V* is a (fixed) symmetric positive definite matrix of size $n \times n$ . The equation (4) can be written equivalently as:

5 $$\begin{aligned} \tau _{(k)} \sim W(\Gamma ,n) \end{aligned}$$

where $\tau _{(k)}= \Sigma _k^{-1}$, is the precision matrix for *k*th region, which is a Wishart prior with degrees of freedom *n* set equal to the number of cancers considered in the model and the scale matrix $\Gamma$ is specified as an identity matrix so that the priors are minimally informative [36].

### Selection of cancer types for multivariate model

The proposed multivariate models are fitted for each of the groups mentioned below. The groups suggested here are suggestive and there could be other possible groupings. In this study, the first two groups are made from generic point of view, grouping the most common cancers and less common and rare cancers in two groups. The third group is chosen from epidemiological context, according to a common risk factor, namely smoking. We acknowledge that cancers can be grouped according to many attributable factors such as: alcohol consumption, UV radiation, insufficient physical activities, hormone etc. [37]. We have included results of the multivariate models fitted to two more potential groups of cancers (hormone related cancers, overweight and obesity related cancers) in Additional file 1.

#### Group 1: Most common cancer types

Among the cancer types reported in ACA, the most common are, prostate, breast, colorectal (bowel), melanoma and lung cancer. These five cancer types account for around 60% of all cancers diagnosed in Australia [38]. To fit the proposed multivariate model, we grouped these common cancer types into subgroups as follows:

- Model 1: Lung, melanoma and bowel cancers : 1(a): for males, 1(b): for females and 1(c): for all persons
- Model 2: Lung, melanoma, bowel and prostate cancers for males
- Model 3: Lung, melanoma, bowel and breast cancers for females

#### Group 2: Less common and rare cancers

According to Cancer Australia, most cancer types, except breast, prostate, bowel, lung and melanoma, can be classified as rare or less common [39]. A rare cancer is defined as a type of cancer that has less than 6 cases per year per 100,000 population, whereas a less common cancer is defined as one that has between 6 and 12 cases per year per 100,000 population [39].

According to the age standardised incidence rates per 100,000 population for Australia for the year 2016, the rare cancer types, among the selected cancer types in ACA, include liver cancer for females (4.7) and oesophageal cancer for females (3.6). The less common cancers include brain cancer (males: 9.1, females: 6.0 and all persons: 7.5), cervical cancer for females (7.1), head and neck cancer for females (8.6), kidney cancer for females (9.4), liver cancer for all persons (8.7), oesophageal cancer for males (8.7) and all persons (6.2), stomach cancer for females (6.4) and all persons (9.3) and thyroid cancer for males (6.5). We created the following subgroups for the less common/rare cancers to fit the proposed model to each group:

- Model 4: Liver and oesophageal cancer for females
- Model 5: Brain, oesophageal and thyroid cancers for males
- Model 6: Brain, Cervical, head and neck, kidney and stomach cancers for females
- Model 7: Brain, liver, oesophageal and stomach cancer for all persons

#### Group 3: Cancers associated with smoking

One of the most studied cancer risk factors is smoking, which has been shown to cause several types of cancer. The following cancers are found to be related to smoking [40–45], which form the last group for fitting the proposed model:

- Model 8: Lung, liver, pancreatic, stomach, kidney, oesophageal and head and neck cancers : 8(a): for males, 8(b): for females and 8(c): for all persons

### Model implementation

A total of 12 multivariate Bayesian meta-analysis models were run for the different combinations of cancer types in R version 3.6.0 [46] using the package R2jags version 0.5-7 [47]. The Markov Chain Monte Carlo (MCMC) model output was summarised in R using the coda package [48]. The JAGS code for the model is given in Appendix A3.

Three parallel MCMC chains, each with 100,000 iterations with a burn in period of 10,000 iterations were run to fit the proposed models. Convergence was examined using visual diagnostics for the parameters of interest $\mu _{ij(k)},\mu _{i(k)}$ and $\Sigma _{k}$.

### Model inferences

From the posterior distributions of the parameters of interest from each of the fitted Bayesian meta-analysis models, the following inferences were drawn in this study.

Comparing the posterior mean *log*(*SIR*) ($\mu _{ij(k)}$) for the group of cancers for each SA2, we identified those SA2s for which all cancers in a group had higher incidence compared to the Australian average.

From the matrix of posterior means, we were able to evaluate the behaviour of a group of cancers in different regions. The posterior covariance matrix for each of the regions was used to obtain the correlation between all possible pairs of cancers in a group within and across different regions (major cities, regional and remote areas). An asymptotic $\chi ^2$ test was used to test the equality of multiple correlation matrices [1].

6 $$\begin{aligned} C^2 = \sum _{i=1}^k \left( \frac{1}{2} tr(Z_i^2) - dg^{'}(Z_i) S^{-1} dg(Z_i)\right) \sim \chi ^2_{(k-1)p(p-1)/2} \end{aligned}$$

where, *tr*(.) denotes the trace of a matrix, $Z_i= \sqrt{n_i} \bar{R}^{-1}(R_i - \bar{R})$, $\bar{R}= (n_ 1R_a + ... + n_k R_k)/n = \bar{r}_{ij}$, $S=(\delta _{ij}+ \bar{r}_{ij} \bar{r}^{ij})$, $\bar{r}^{ij}= \bar{r}_{ij}^{-1}$, $R_1, R_2, ..., R_k$ are sample correlation matrices based on k independent samples of sizes $n_1,n_2, ...,n_k$ from $p-$variate normal populations, $\delta _{ij}$ is the Kronecker delta and $dg(Z_i)$ denotes the diagonal of a square matrix $Z_i$ of correlation coefficients.

Using Jennrich’s test, we identified which cancers had substantially different correlation matrices in urban, regional and remote Australia.

Using the model inferences, high risk areas for each cancer and the groups of cancer types are identified. High risk areas are defined as the SA2s having an SIR likely to be greater than one, which means the incidence rate for that area is higher than among the reference population (the Australian national average). Several options are possible to identify the areas, but here posterior probabilities (PPs) are used. The PP that an estimated SIR of a particular cancer is greater than the national average can be calculated for each SA2. It is defined as the ratio of the number of MCMC iterations in which the modelled SIR is above 1, divided by the the total number of iterations [19]. SA2s with PP $\ge 0.80$ can be considered as a high risk area for a cancer [49]. An area with high risk for more than one cancer (high PP for SIR greater than 1) was defined as high risk for multiple cancers. In contrast, the low risk areas for a group of cancer types are defined as the SA2s where none of the cancer types in the group is defined as being high risk.

## Results

**Fig. 1:**
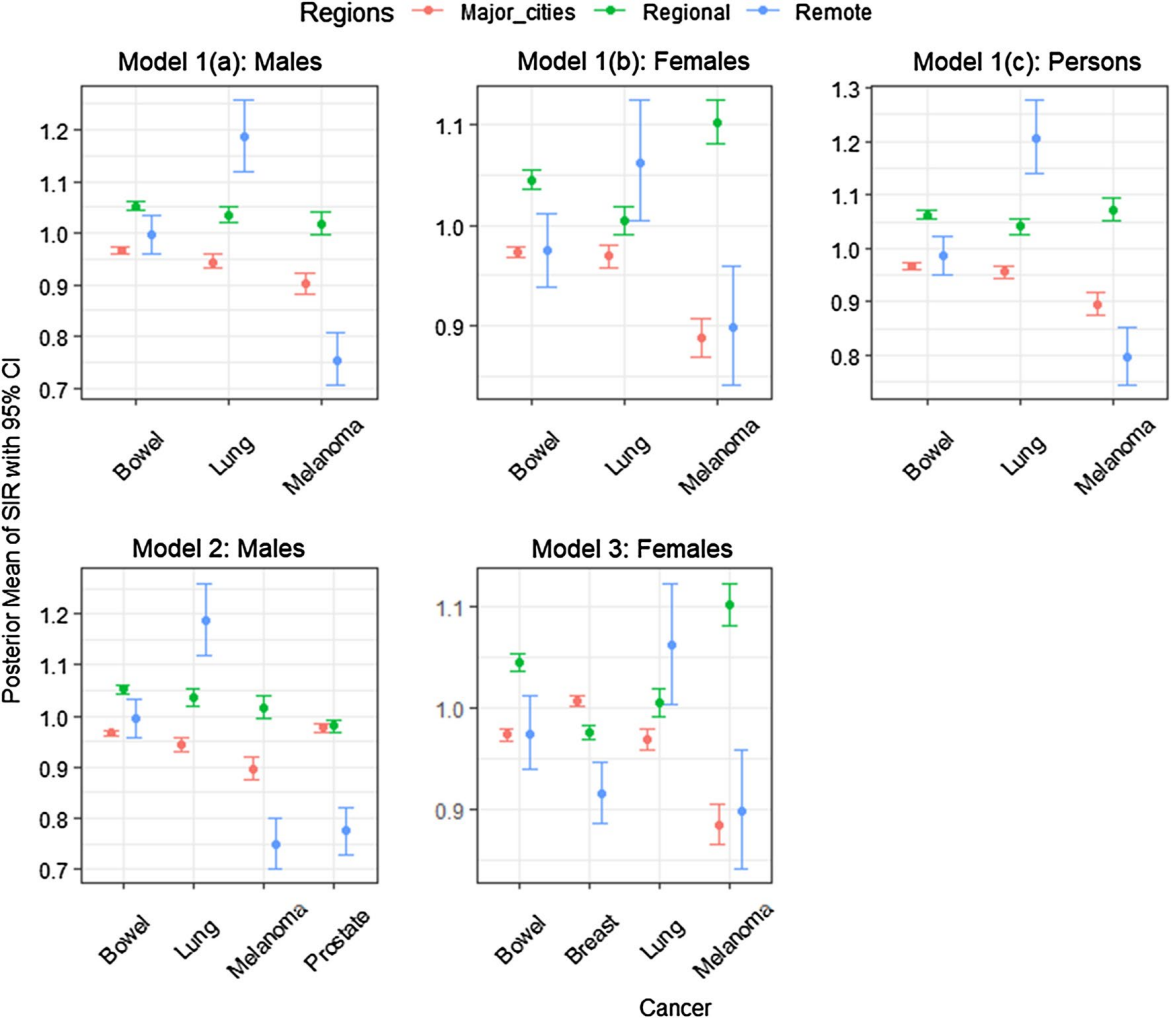
Posterior means with 95% credible intervals of SIR for the most common cancers (Group 1) over remoteness regions, Australia

**Fig. 2:**
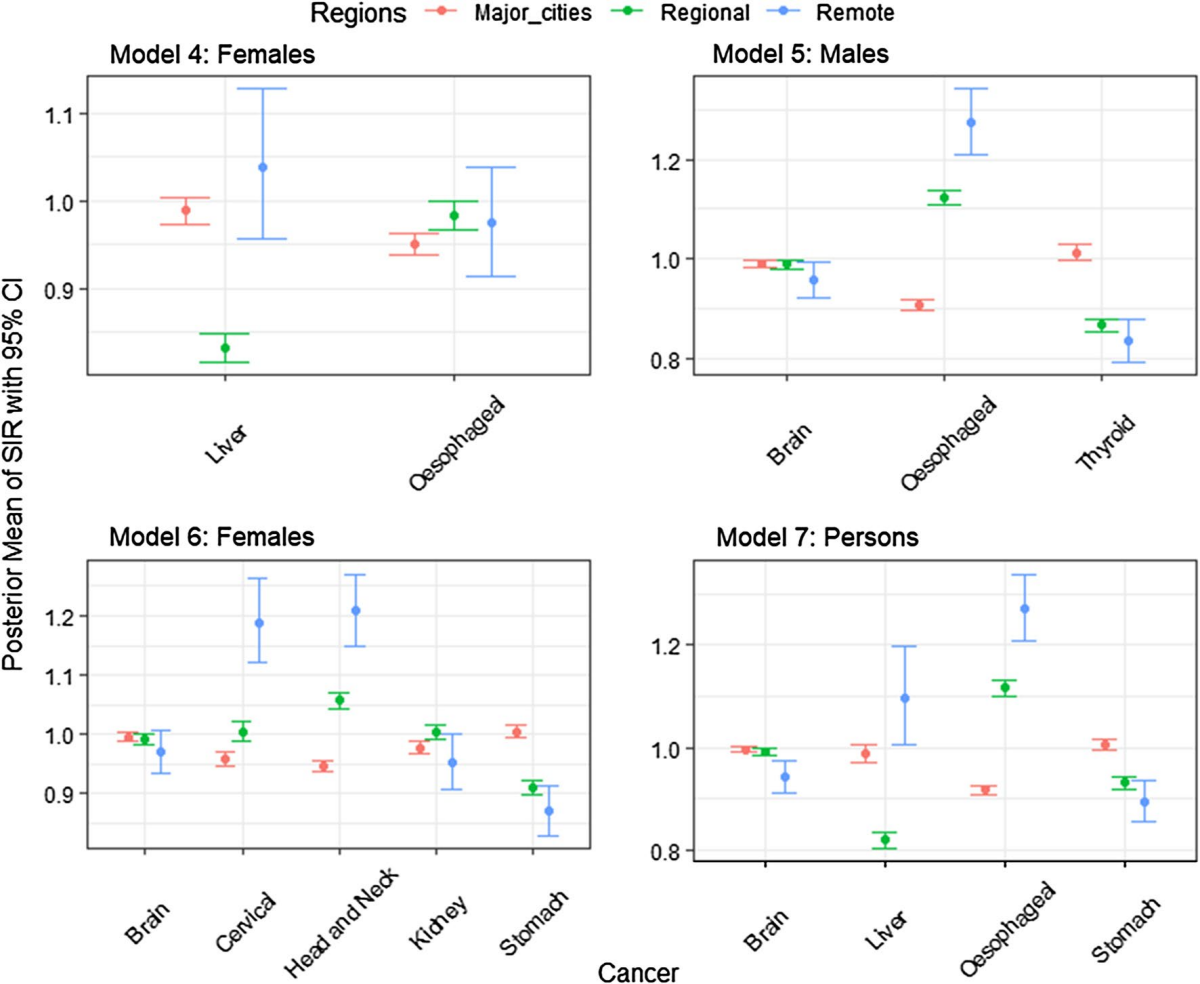
Posterior means and 95% credible intervals of SIR for the less common cancers/rare cancers (Group 2) over remoteness regions, Australia

**Fig. 3:**
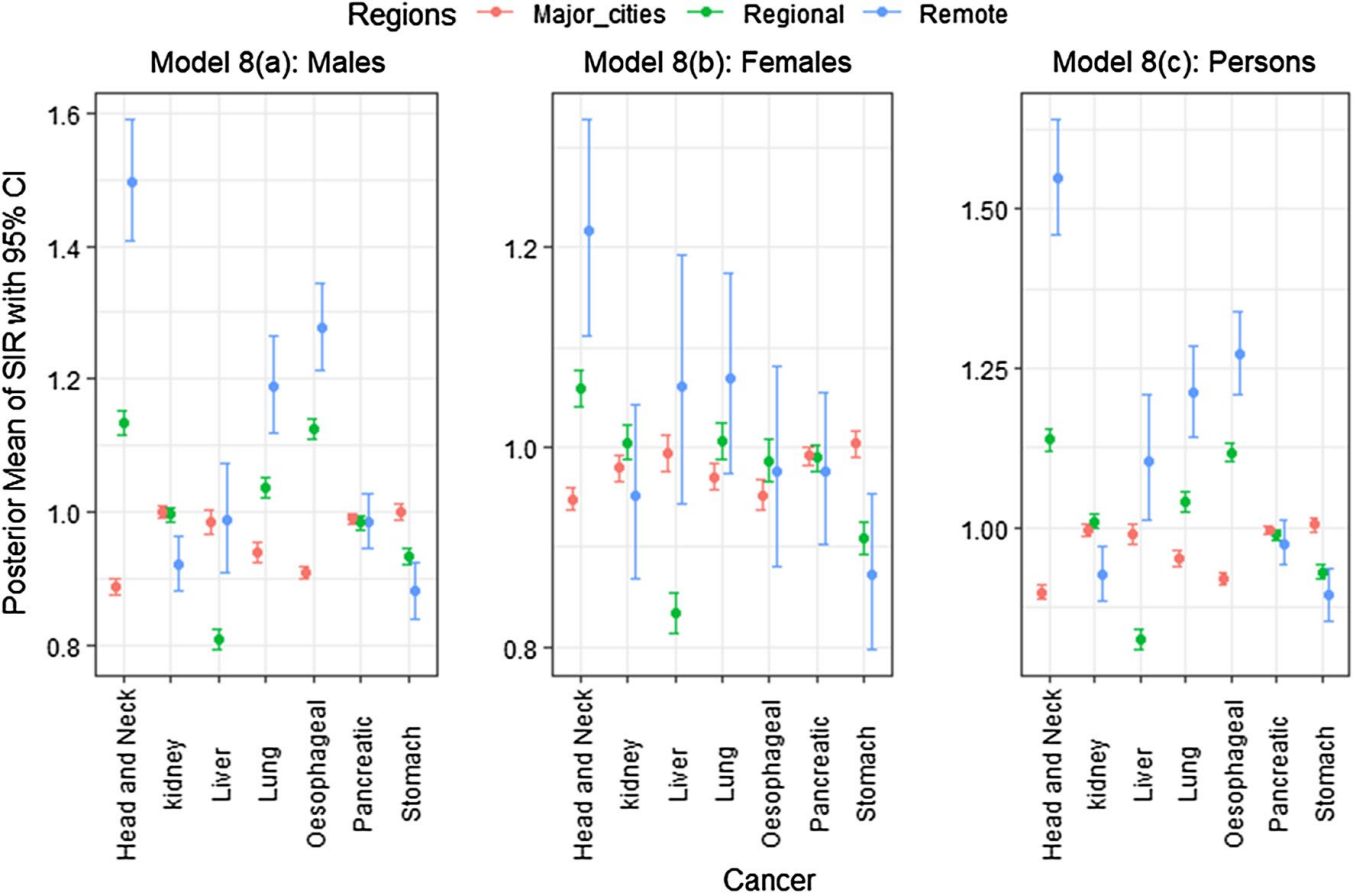
Posterior means and 95% credible intervals of SIR for the smoking related cancers (Group 3) over remoteness regions, Australia

The posterior means with 95% credible interval for $\mu _{i(k)}$ of each group of cancer types in each of the 3 remoteness categories (namely major cities, regional and remote areas) under each of the 12 models are shown in Figs. 1, 2 and 3 (for the actual values of posterior means, see Additional file 1).

Figures 1, 2 and 3 demonstrate how different cancers have different incidence patterns over different regions of Australia. For example, Fig. 1, the highest melanoma incidence has occured in regional areas, whereas lung cancer has higher incidence in remote areas (for males, females and all persons). Figure 2 (Model 6), remote areas had the highest incidence of cervical and head and neck cancers among all persons on average.

**Fig. 4:**
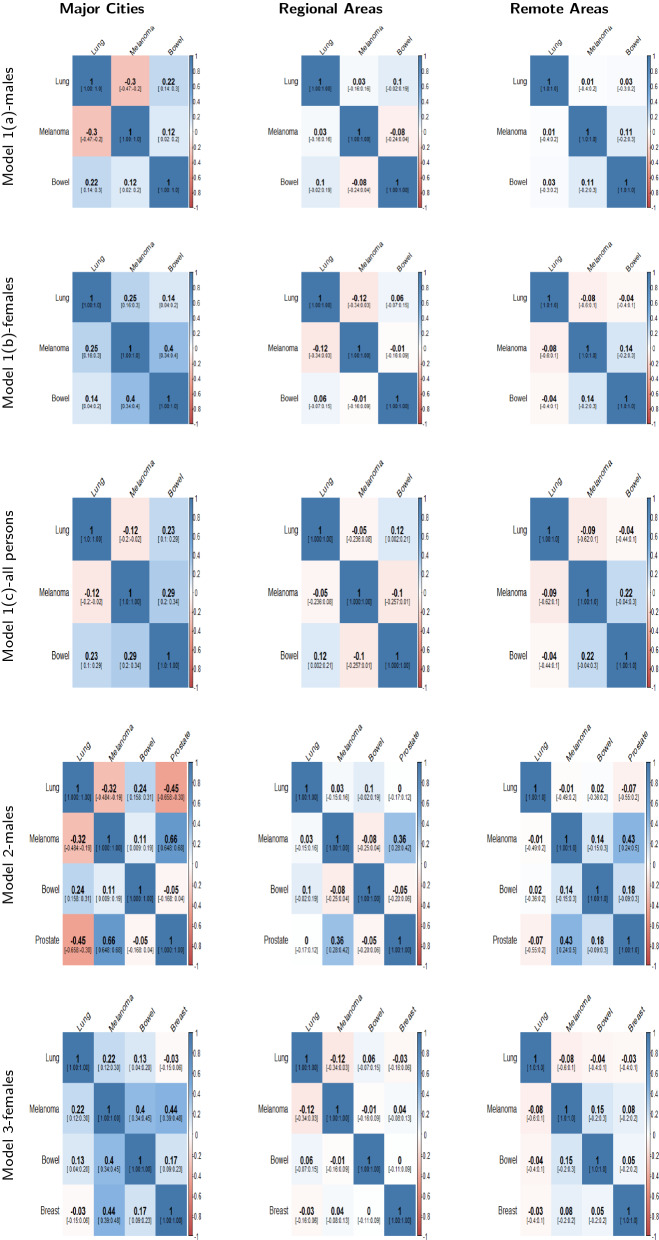
Posterior Correlation matrices with 95% credible intervals for most common cancers (Model 1(a,b,c),2 & 3) by region

**Fig. 5:**
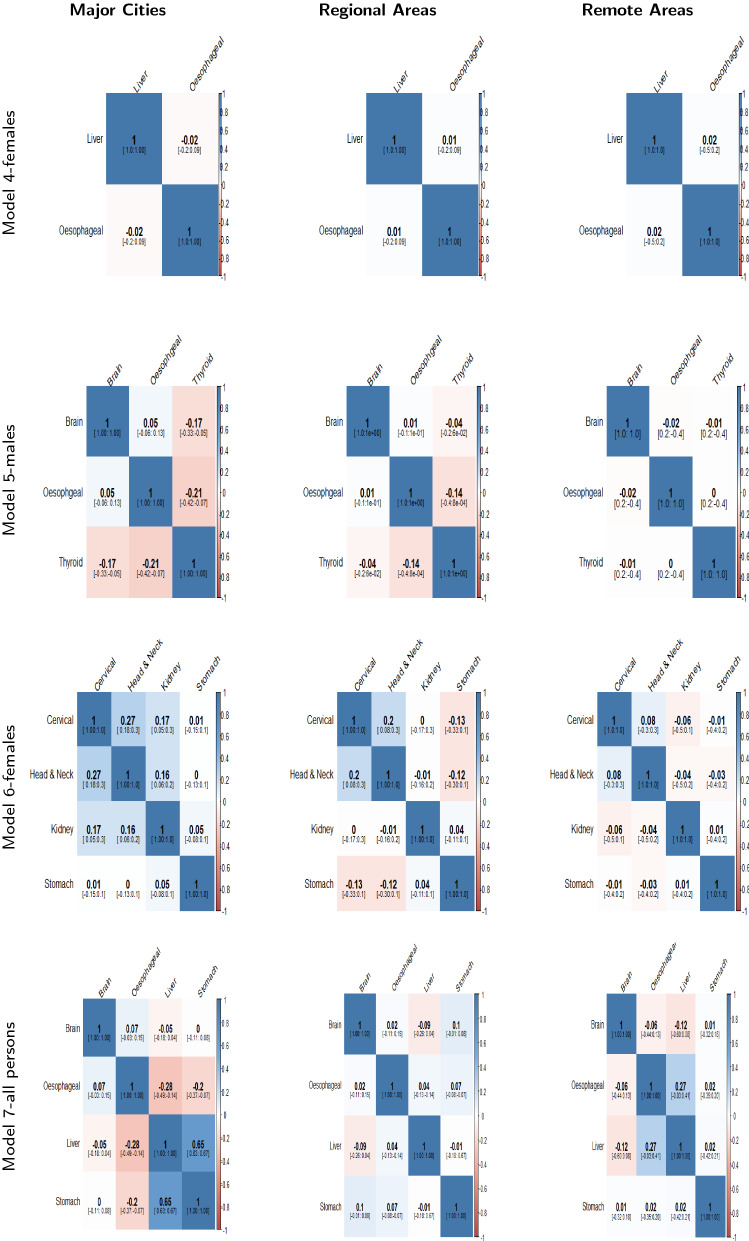
Posterior Correlation matrices with 95% credible intervals for smoking related cancers (Model 4,5,6,& 7) by region

**Fig. 6:**
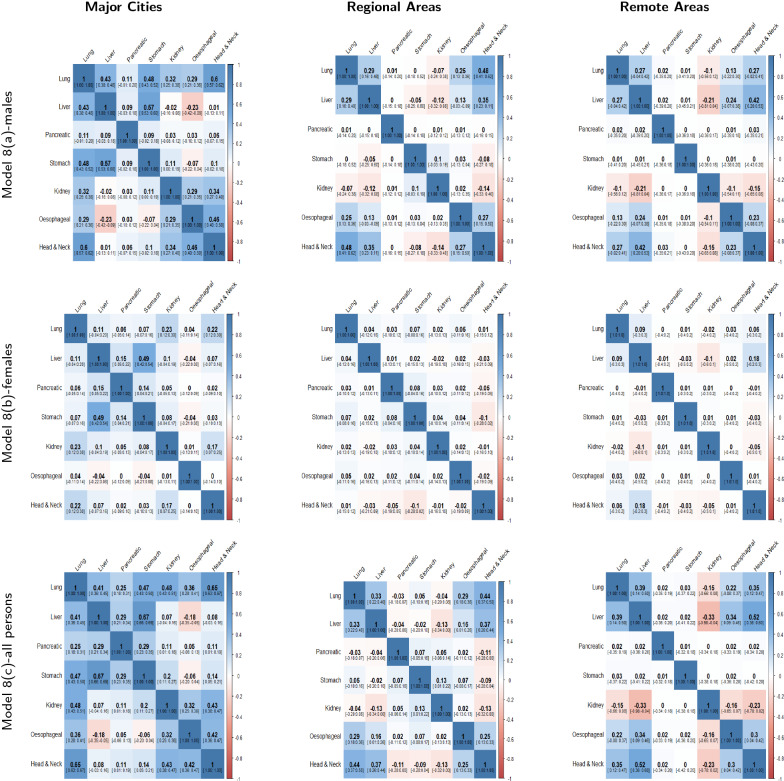
Posterior Correlation matrices with 95% credible intervals for smoking related cancers (Model 8) by region

The mean posterior correlation matrices for each model in the three different regions are shown in Figs. 4, 5 and 6. When two cancers have positive correlation, it means that incidence patterns for both cancer types are similar in that particular region. If the $95\%$ credible interval of the correlation coefficient includes zero, it is assumed that no substantive correlation is present between the incidence patterns of the pair of cancer types under consideration.

The posterior correlation matrices for most common cancer types (Models 1a, 1b, 1c, 2 & 3) are presented in Fig. 4. In Fig. 4, we can see that the correlation coefficients of melanoma and lung cancer are negative in major cities (for males: model 1(a) and all persons: model 1(c)) and these are not substantially correlated in regional and remote areas. Some more examples of correlation in different regions: negative correlation between prostate and lung cancers in cities and no correlation in regional and remote areas (Fig. 4: Model 2) and significant positive correlation between breast cancer and melanoma in cities and no substantive correlation in regional and remote areas (Fig. 4: Model 3).

In Fig. 5, the posterior correlation matrices for rare and less common cancers are reported. We can observe no substantive correlation between liver and oesophageal cancer for females in all three regions (Model 4). Thyroid and brain cancer have negative correlation in major cities but no correlation in regional and remote areas (Model 5). Head and neck cancer and cervical cancer have a significant positive correlation in major cities and regional areas but none in remote areas (Model 6).

Figure 6 shows the $7 \times 7$ correlation matrices for smoking related cancers for males, females and all persons (Model 8a, 8b and 8c). As can be seen, correlation can substantially differ between the same pairs of cancers across major cities, regional and remote areas. For example, stomach and lung cancers have significant positive correlations for males and all persons (model 8a and 8c) in major cities but there is no substantive correlation between these cancers in regional and remote areas. Similarly, lung and kidney cancers for males, females and all persons (Model 8a, 8b and 8c) have significant positive correlation in major cities and weak or no correlation in regional and remote areas. There are also similar correlations across different regions among pairs of cancers. For instance, lung, head and neck cancers are positively correlated in major cities, regional and remote areas for all persons (Model 8c).

Clearly, different models have some similarities and dissimilarities according to pairwise correlation. From Jennrich’s test (Table 1), substantive differences among the correlation matrices were found for the majority of models including most common cancers (Models 1a, 1b, 1c, 2 & 3), rare and less common cancers (Models 6 & 7) and smoking related cancers (Models 8a, 8b & 8c) (Table 1). For the other less common/rare cancers (Models 4 and 5) care should be taken due to small sample sizes.

**Table 1:** Results of Jennrich’s Test of differences in Correlation matrices applied to each group of cancers in different remoteness regions

Group	Model	Test Statistic $^{\mathrm{a}}$	P value
Most common cancers	1(a)	114.74	< 0.0001
	1(b)	155.91	< 0.0001
	1(c)	111.42	< 0.0001
	2	282.87	< 0.0001
	3	250.55	< 0.0001
Less common and rare cancers	4	0.65	0.7225
	5	2.68	0.8481
	6	44.31	< 0.0001
	7	384.79	< 0.0001
Smoking related cancers	8(a)	767.74	< 0.0001
	8(b)	226.35	< 0.0001
	8(c)	1005.75	< 0.0001

$^{\mathrm{a}}$ Null Hypothesis: Equality of correlation matrices in major cities, regional and remote areas for each group of cancers are tested

**Fig. 7:**
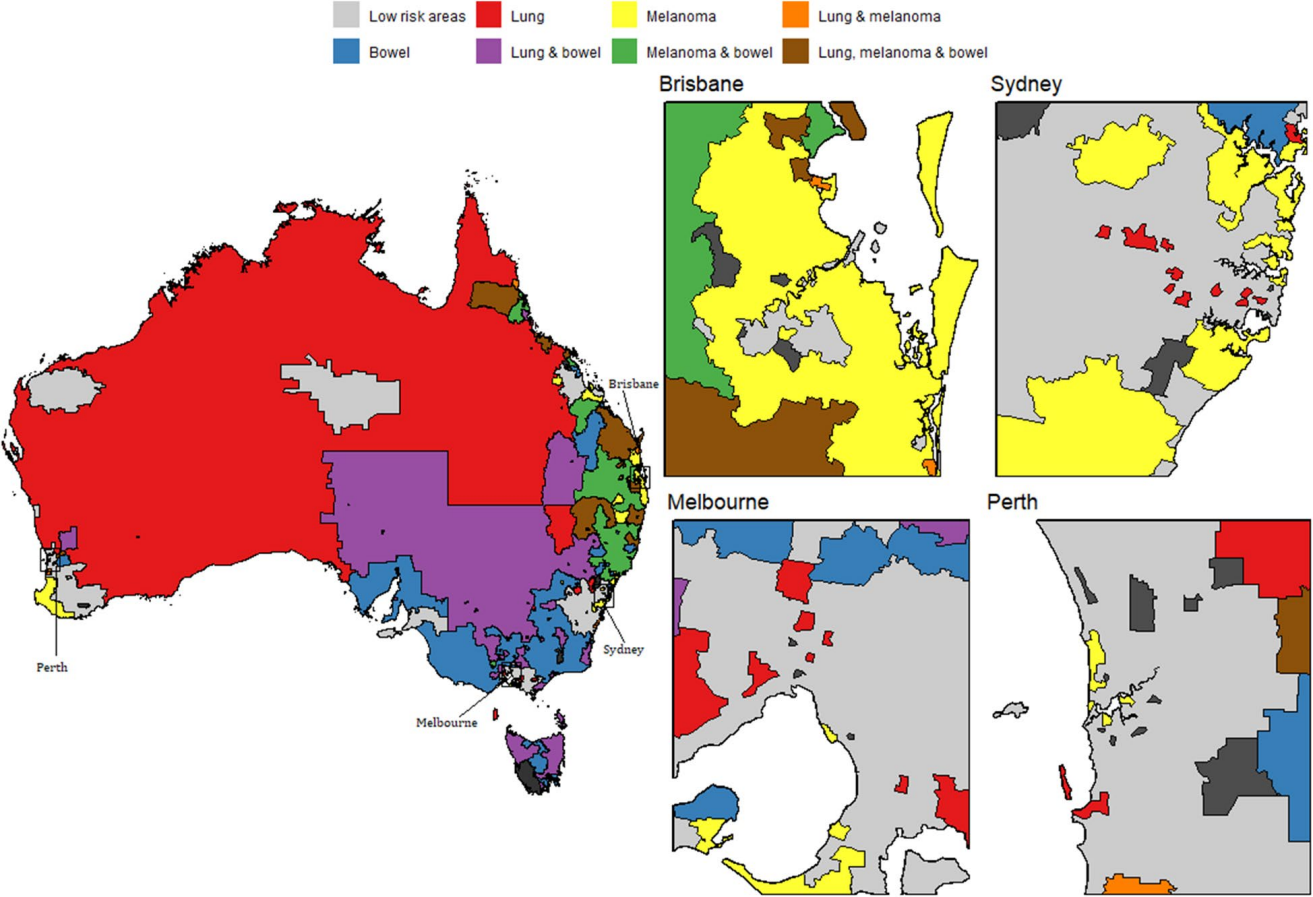
High and low risk areas for individuals and multiple cancers (most common cancers for all persons: Model 1(c))

**Fig. 8:**
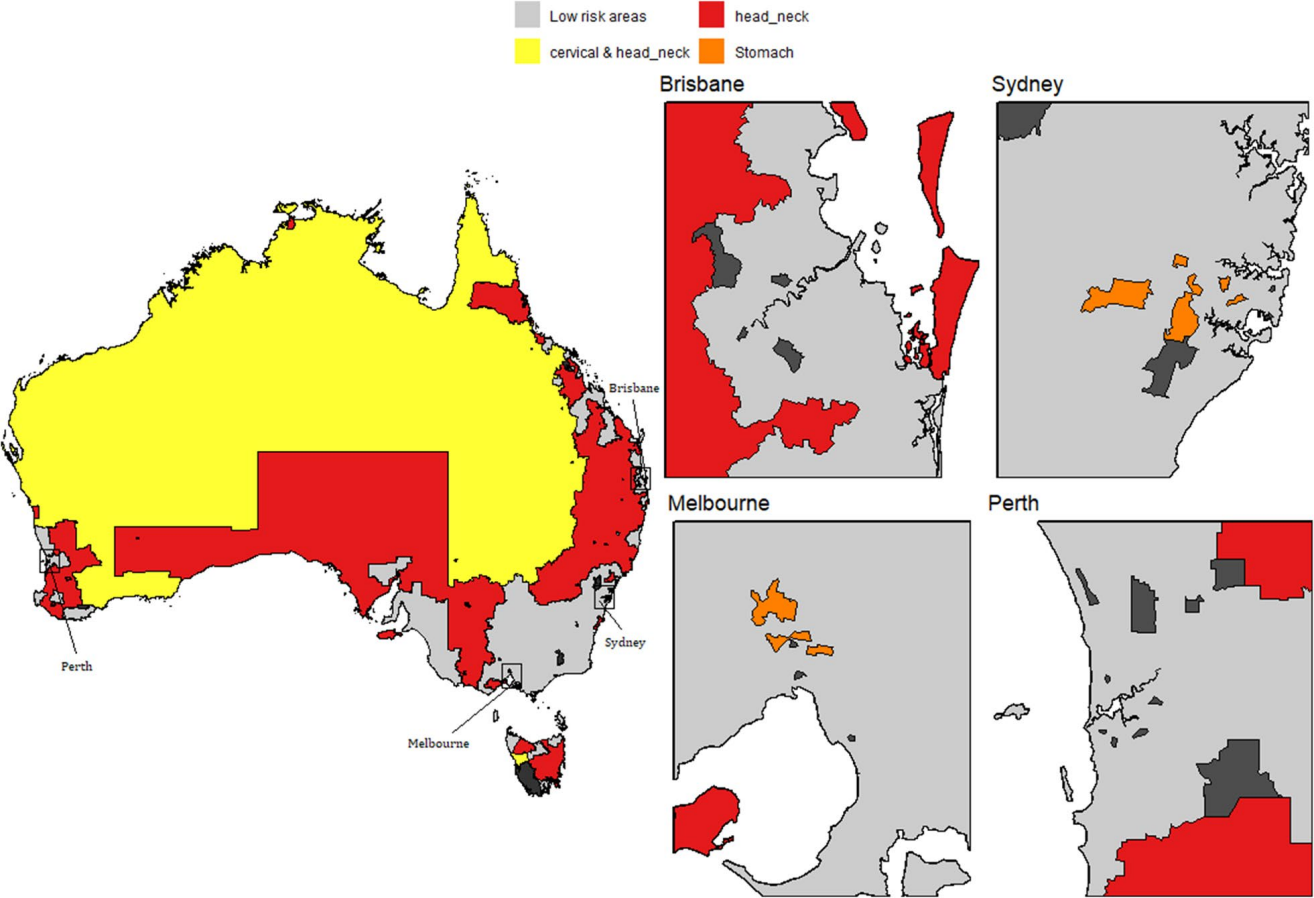
High and low risk areas for individuals and multiple cancers (less common/rare cancers for females: Model 6)

**Fig. 9:**
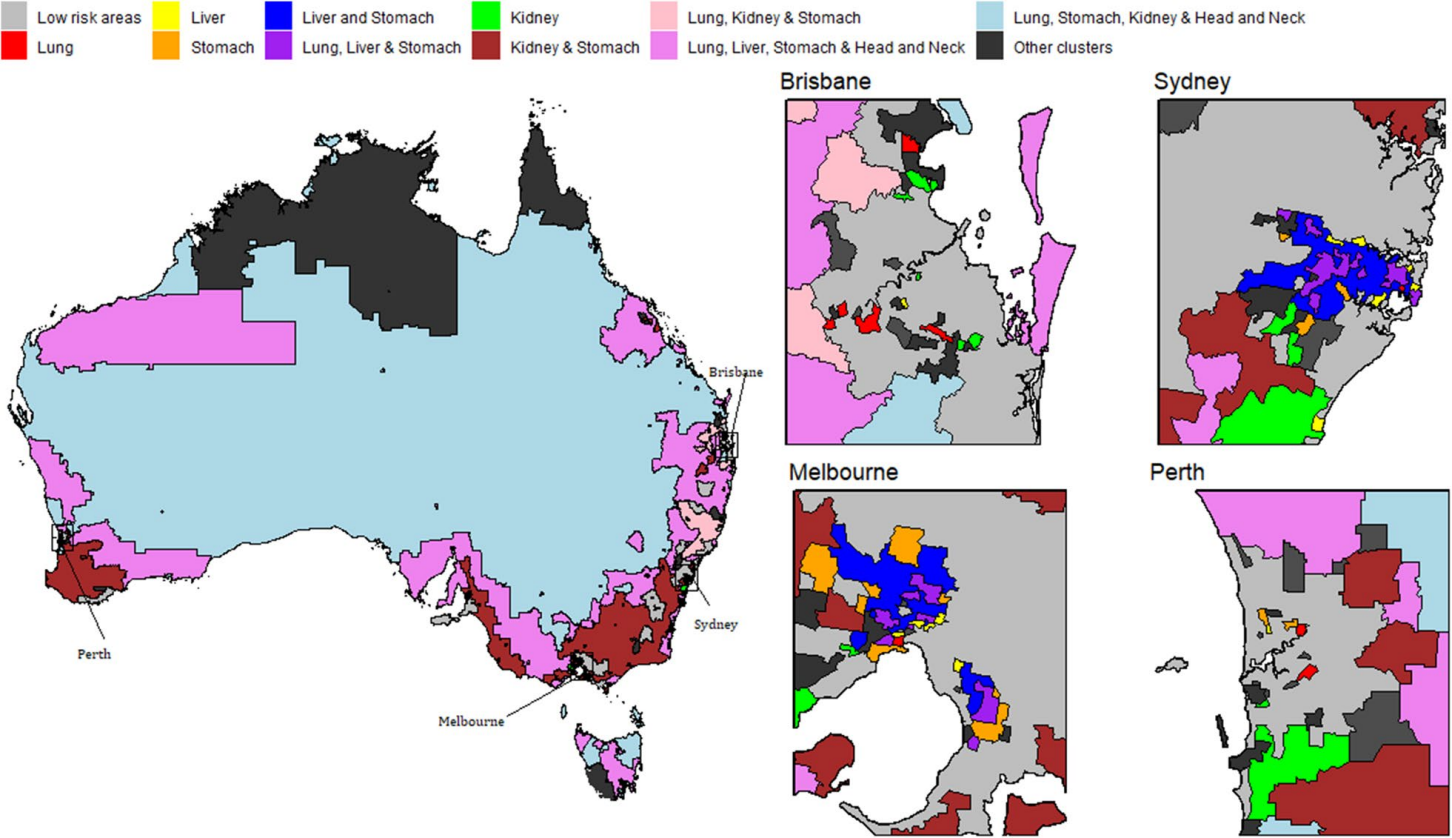
High and low risk areas for individuals and multiple cancers (smoking related cancers for males: Model 8(a))

**Fig. 10:**
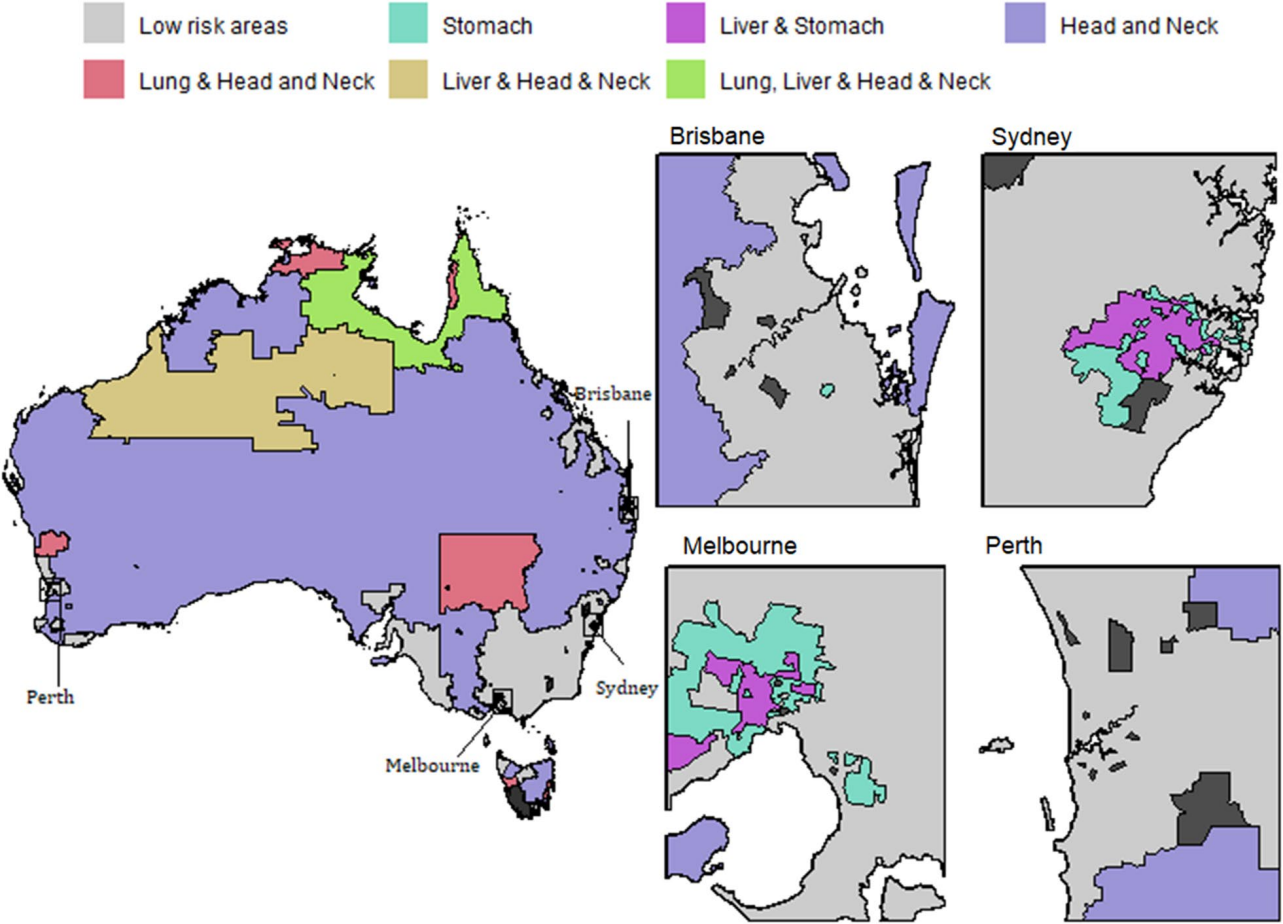
High and low risk areas for multiple cancers (smoking related cancers for females: Model 8(b))

Using the model inferences, we have identified small areas (SA2s) with higher incidence of a cancer or a group of cancer types; namely high risk areas. Figs. 7, 8, 9 and 10 show some examples of high-risk areas for each group of cancer types around Australia. For most common cancers (group 1, model 1), the spatial map in Fig. 7 shows high risk areas for each cancer type of the group individually as well as jointly, (lung, melanoma and bowel individually; lung and melanoma; lung and bowel; lung, melanoma and bowel jointly, for all persons, Model 1c). While identifying an area with high risk for multiple cancers in a group, for example, an area was identified as high risk areas for both lung and melanoma are those areas which were identified as high risk areas (have higher PP for estimated SIR to be greater than 1) for both lung and melanoma cancer types. The cluster of areas having high risk for group of cancers are also identified similarly under each model (see Figs. 7, 8, 9 and 10 and Tables 2 and 3). To enable a clearer view, four insets of the full map are shown alongside. A map of Australia with locations of states and capitals of each state in Australia is shown in Additional file 1: Figure S16). This map is intended to help the readers interpreting the spatial maps visualising high and low risk areas for groups of cancers.

**Table 2:** Number of SA2s with higher incidence for groups of cancers jointly and individually, models 19(c) and 6

Group	Cancer	No. of SA2s
Most Common Cancers (for all persons): Model 1(c)	Lung only	143
	Melanoma only	442
	Lung & melanoma	22
	Bowel only	238
	Lung & bowel	123
	Melanoma & bowel	116
	Lung, melanoma & bowel	76
Less Common/ Rare Cancers (for females): Model 6	Head & neck only	378
	Cervical & Head and neck	79
	Stomach only	13

From Table 2, we can see 76 SA2s out of 2148 are identified as high risk areas for all three cancers considered in Model 1 (for all persons). There are 22 SA2s around Australia which have higher incidence for lung and melanoma, 123 SA2s for lung and bowel, 116 SA2s for melanoma and bowel jointly. For more information on the number of SA2s having substantially higher SIRs for individual and joint cancers under selected models, refer to Tables 2 and 3. Only four models out of the twelve are illustrated to show the high risk areas in maps in this section. In Fig. 9, only groups of cancer types with 20 or more SA2s are showed in the map. The groups of cancers with less than 20 areas in each group are combined as other clusters. For more details of the groups included in other clusters, please see Table 3.

## Discussion

A multivariate Bayesian meta-analysis model was proposed in the present study to model multiple cancers jointly to identify any existent relationships among the cancers. The advantages of this model include that it incorporates the uncertainty of the modelled summary estimates, it allows for easy identification and visualisation of areas with high risk for different combinations of cancer types, and it is readily extendable.

The proposed model was illustrated by joint modelling of multiple cancers in different groups formed from the 20 cancers included in the ACA. The most common cancers (Models 1,2,3) and the smoking related cancers (Model 8) were found to have significantly different correlation matrices across major cities, regional and remote areas. These findings imply that additional factors influencing cancer incidence in the three different regions may be present. Some of the cancers could be associated with other environmental and socio-economic factors which could be different in different regions. Among the less common and rare cancers group, models 6 and 7 have a significantly different correlation matrix in each of the three regions. The correlation coefficients in each of the correlation matrices represent the correlation between incidences of pairs of cancer types within each cancer group and region.

Mostly in the published literature, multivariate meta-analyses of cancer have focused on exploring the relationship between risk factors/prognostic factors and specific cancers [42, 50–54]. The present study is the first of its kind identifying correlation between the incidence of pairs of cancer types in selected groups. While some of the obtained results support the already known facts, some of the results are new and could create opportunities for further investigation into the reasons for the observed patterns. For instance, the smoking related cancers are modelled jointly (Model 8) for males, females and all persons. These cancers are expected to have positive correlation, yet significant negative correlation was observed between oesophageal and liver cancer incidence (for males & all persons in major cities) as well as kidney, head and neck cancers (for all persons in remote areas). It may be that these cancers are predominantly driven by risk factors that could not be included in our analysis, such as obesity (for oesophageal cancer) or chronic hepatitis viral infection (for liver cancer). These models can be used to identify unexpected negative correlations for further investigation.

The proposed multivariate Bayesian hierarchical meta-analysis model is applied to model the publicly available smoothed estimates of multiple cancers jointly. Such an approach is useful when the raw data are unavailable and can be used to answer additional research questions of interest. In the present scenario, we do not consider multiple testing as a limitation of this study since the various inferences were derived from the full joint posterior distributions as is appropriate under the Bayesian paradigm. In terms of the tests for equality of correlation matrices, only one hypothesis test was undertaken for each multivariate model and each model had different groups of cancers, hence a different dataset. However, when applying these models to groups of cancers, one consideration is that the choice of cancers can have a noticeable impact on estimates obtained (see  Additional File 1). For instance, in Fig. 1, Melanoma for females has slightly different estimates in model 1(b) and model 3. This results from the multivariate nature of the proposed model and the covariance structure within the group. Since correlation is a standardised form of covariance, the precision of estimates and the correlation between cancers are related. Since the choice of cancer types included may influence the results, we recommend comparing the multivariate results with the univariate results (using an approach such as [32]). Also, because this model was developed for summarised modelled estimates the proposed model cannot be applied to raw incidence rates without modifying, such as introducing some form of spatial smoothing and changing the distributional assumptions at different levels.

The hierarchy introduced in the model using the remoteness structure of each of the small areas could also be replaced by any other factor of interest. For example, in the present model, if we wanted to check how the correlation among the multiple cancers differs in different states across Australia, we could use states as the hierarchy instead of the remoteness regions. The hierarchical stage could also be extended in a straightforward manner to include more than one factor. For example, we could use both states and regions in the model by including one more hierarchy in the existing model. We could also extend the model by using socio-economic status of each area as another factor of interest.

Although the proposed model is illustrated for exploring the relationship of multiple cancers in different remoteness regions in Australia, this approach can be used in a straightforward manner for any other cancer atlases from any country or region. The approach provides a multivariate Bayesian meta-analysis model framework that can combine multiple outcomes from any available online sources where summary measures are available. For instance, the Atlas of Cancer Mortality in the European Union and the European Economic Area, 1993-1997 [55], provides estimates of age standardised mortality rates of 30 different cancer types in 1278 small areas in 28 different member countries of EU. A similar approach can be taken to model the multiple cancers jointly and a possible hierarchy could be the different countries (members of EU) to identify any patterns.

**Table 3:** Number of SA2s with higher incidence of Smoking related cancers (for males), Model 8(a) jointly and individually

Group	Cancer	No. of SA2s
Model 8(a): for males	Low risk areas	990
	Lung	21
	Liver	24
	Lung & Liver	1
	Stomach	20
	Lung & Stomach	11
	Liver and Stomach	113
	Lung, Liver & Stomach	51
	Kidney	26
	Lung & Kidney	17
	Kidney & Stomach	2
	Lung, Kidney & Stomach	5
	Lung, Liver, Stomach & Kidney	10
	Oesophageal	200
	Lung and Oesophageal	1
	Head & Neck	36
	Lung & Head and Neck	7
	Lung, Liver, Stomach & Head and Neck	1
	Lung, Kidney & Head and Neck	13
	Lung, Stomach, Kidney & Head and Neck	2
	Lung, Liver, Stomach, Kidney & Head and Neck	1
	Oesophageal & Head and Neck	294
	Lung,Oesophageal & Head and Neck	284
	Lung, Liver, Oesophageal & Head and Neck	18
Model 8(b): for females	Stomach only	98
	Liver & Stomach	71
	Head and Neck only	434
	Lung & Head and Neck	14
	Liver& Head and Neck	5
	Lung, Liver & Head and Neck	5

## Conclusions

This study presents a novel use of Bayesian meta-analysis for multivariate modelling of reported cancer incidence estimates. The modelling technique can be generalised for other disease maps or atlases. The proposed modelling approach is flexible for joint modelling of multiple estimated disease outcomes with different research questions of interest. The scope for this model is vast, and we anticipate it being a useful addition for analysing summary estimates in more detail.

### Electronic supplementary material

**Additional file  1.** Additional material of Multivariate Bayesian meta-analysis: joint modelling of multiple cancers using summary measures. More results from the proposed model in form of tables and figures are provided in this additional file.

## Data Availability

The cancer incidence data used in this research can be downloaded from the Australian Cancer Atlas website (https://atlas.cancer.org.au/). The other data set used in this research is the remoteness indexes which can be downloaded from ABS ASGS 2011 website (https://data.gov.au/dataset/ds-dga-4b208cc1-f5de-405d-af96-0777645dfc87/details?q=). Some transformations are made to get the remoteness indexes for each of the SA2s.

## Appendices

### A1. Cancers included in ACA by sex

**Table:**

Cancer	International Classification of Diseases (ICD)-10 codes	Description	Sex
All	C00–C97, D45, D46, D47.1, D47.3–D47.5	All malignant cancers combined	Males, Females, all persons
Bowel	C18-–C20	Inner lining of the large bowel (colon) or rectum.	Males, Females, all persons
Brain	C71	Brain. Excluding cranial nerves and retrobulbar tissue	Males, Females, all persons
Breast	C50	Breast (excluding skin)	Females
Cervical	C53	Lining of the cervix	Females
Head and Neck	C00–C14, C30–C32	Tissue or lymph nodes in the head and neck area	Males, Females, all persons
Kidney	C64	Kidney. Except renal pelvis	Males, Females, all persons
Leukaemia	C91-C95	White blood cells of the bone marrow	Males, Females, all persons
Liver	C22	Liver and intrahepatic bile ducts	Males, Females, all persons
Lung	C33-C34	Lung, trachea, bronchus	Males, Females, all persons
Melanoma	C43	Melanocytes (pigmented cells) of the skin	Males, Females, all persons
Myeloma	C90	Plasma cells of the bone marrow	Males, Females, all persons
Non-Hodgkin Lymphoma	C82-C86	Lymphatic system	Males, Females, all persons
Oesophageal	C15	Oesophagus	Males, Females, all persons
Ovarian	C56	Ovaries	Females
Pancreatic	C25	Pancreas	Males, Females, all persons
Prostate	C61	Prostate gland	Males
Stomach	C16	Lining of the upper part of the stomach	Males, Females, all persons
Thyroid	C73	Thyroid gland	Males, Females, all persons
Uterine	C54-C55	Lining of the uterus, the muscle or the connective tissue	Females

### A2. More hierarchy in the multivariate meta-analysis model

The possible hierarchical modelling after equation (2) is shown below using the usual Bayesian hierarchical framework below. Although the parameters therein are not of interest for this particular case study, it can be appropriate for other types of data and/or research questions.

7 $$\begin{aligned} \mu _{i(k)} \sim MVN(\mu _{i}, \omega _i) \end{aligned}$$

where $\mu _i$ is the overall mean of $i{th}$ cancer and $\omega _i$ be the variance covariance term accounting for variation between the means of different regions and same cancers. We are not interested in these parameters as we already performed univariate analysis to see the means and variation due to remoteness for each cancer separately [32].

The overall mean of $i{th}$ cancer, $\mu _i$ can then be modelled as:

8 $$\begin{aligned} \mu _{i} \sim N(\mu _0, \sigma _0^2) \end{aligned}$$

where, $\mu _0$ is the overall mean of all cancers in Australia, and $\sigma _0^2$ is the variance among the means of different cancers.

We can choose to expand the model differently as well. Instead of modelling each cancer separately, we can choose to model mean of cancer incidence in each region separately as:

9 $$\begin{aligned} \mu _{i(k)} \sim MVN(\mu _{k}, \omega _k) \end{aligned}$$

where $\mu _k$ is the overall mean of the $k{th}$ region and $\omega _k$ is the variance covariance term accounting for variation between the means of different cancers and same regions. The above equation could be used instead of equation (7) and the equation (8) could be updated accordingly. The choice of model representation depends on which parameters we are of interest and the desired inferences.
