# Supplementary material for: Multivariate Bayesian meta-analysis: joint modelling of multiple cancer types using summary statistics
Source: Int J Health Geogr. 2020 Oct 17;19:42. doi: 10.1186/s12942-020-00234-0 (PMC7568363; doi:10.1186/s12942-020-00234-0)
Supplement: Supplementary file 1 — Additional file 1. Additional material of Multivariate Bayesian meta-analysis: joint modelling of multiple cancers using summary measures. More results from the proposed model in form of tables and figures are provided in this additional file. [file 12942_2020_234_MOESM1_ESM.pdf]

ADDITIONAL FILE

# Additional File of Multivariate Bayesian meta-analysis: joint modelling of multiple cancers using summary measures

Farzana Jahan[1*], Earl W. Duncan[1], Susana M. Cramb[2], Peter D. Baade[3] and Kerrie L. Mengersen[1]

*Correspondence:
f.jahan@hdr.qut.edu.au
[1]ARC Centre of Excellence in Mathematical and Statistical Frontiers, School of Mathematical Sciences, Science and Engineering Faculty, Queensland University of Technology, QLD 4001 Brisbane, Australia
Full list of author information is available at the end of the article

## Posterior Summary of region specific mean $log(SIR)$ and maps visualising high risk areas for Multivariate Models

The posterior means and 95% credible intervals for region specific means of $log(SIR)$ for each of the fitted multivariate Bayesian meta-analysis models are shown Tables S1-S3.

Using each of the 12 multivariate models, the high risk areas (SA2s) for specific groups of cancers are identified and plotted in the spatial maps (Figures S1-S9). High risk areas are defined as the SA2s having an SIR substantially larger than the Australian average using the posterior probabilities (SA2 having PP $\geq$ 0.80, that a cancer in the SA2s has SIR more than the Australian average 1). The number of areas identified to be high risk areas under each group of cancers are also identified and mentioned in Tables S4-S6. Figure S16 shows a map of Australia with locations of states and capitals of each state in Australia. This map is intended to help the readers interpreting the spatial maps visualising high and low risk areas for groups of cancers.

Table S1: Posterior summary of region specific mean $log(SIR)$ for most common cancers

| Model | Cancer | Region | Mean | 2.5% quantile | 97.5% quantile |
|---|---|---|---|---|---|
| Model 1(a): Males | Bowel | Major Cities | -0.034 | -0.037 | -0.026 |
| | | Regional | 0.051 | 0.042 | 0.059 |
| | | Remote | -0.004 | -0.042 | 0.034 |
| | Lung | Major Cities | -0.057 | -0.071 | -0.043 |
| | | Regional | 0.035 | 0.020 | 0.051 |
| | | Remote | 0.171 | 0.111 | 0.230 |
| | Melanoma | Major Cities | -0.103 | -0.126 | -0.079 |
| | | Regional | 0.018 | -0.004 | 0.040 |
| | | Remote | -0.283 | -0.349 | -0.216 |
| Model1(b): Females | Bowel | Major Cities | -0.027 | -0.033 | -0.021 |
| | | Regional | 0.044 | 0.036 | 0.053 |
| | | Remote | -0.026 | -0.063 | 0.012 |
| | Lung | Major Cities | -0.031 | -0.043 | -0.020 |
| | | Regional | 0.005 | -0.009 | 0.019 |
| | | Remote | 0.060 | 0.004 | 0.118 |
| | Melanoma | Major Cities | -0.119 | -0.141 | -0.097 |
| | | Regional | 0.098 | 0.078 | 0.117 |
| | | Remote | -0.107 | -0.173 | -0.0414 |
| Model 1(c): Persons | Bowel | Major Cities | -0.034 | -0.042 | -0.028 |
| | | Regional | 0.060 | 0.052 | 0.068 |
| | | Remote | -0.014 | -0.051 | 0.022 |
| | Lung | Major Cities | -0.046 | -0.059 | -0.033 |
| | | Regional | 0.040 | 0.026 | 0.054 |
| | | Remote | 0.187 | 0.130 | 0.246 |
| | Melanoma | Major Cities | -0.111 | -0.134 | -0.087 |
| | | Regional | 0.069 | 0.049 | 0.089 |
| | | Remote | -0.229 | -0.297 | -0.161 |
| Model 2: Males | Bowel | Major Cities | -0.034 | -0.041 | -0.028 |
| | | Regional | 0.051 | 0.042 | 0.059 |
| | | Remote | -0.005 | -0.042 | 0.034 |
| | Lung | Major Cities | -0.059 | -0.073 | -0.045 |
| | | Regional | 0.035 | 0.020 | 0.05 |
| | | Remote | 0.172 | 0.111 | 0.231 |
| | Melanoma | Major Cities | -0.109 | -0.132 | -0.085 |
| | | Regional | 0.016 | -0.006 | 0.038 |
| | | Remote | -0.288 | -0.356 | -0.222 |
| | Prostate | Major Cities | -0.023 | -0.033 | -0.014 |
| | | Regional | -0.020 | -0.033 | -0.008 |
| | | Remote | -0.256 | -0.315 | -0.197 |
| Model 3: Females | Bowel | Major Cities | -0.027 | -0.033 | -0.021 |
| | | Regional | 0.044 | 0.036 | 0.053 |
| | | Remote | -0.025 | -0.062 | 0.012 |
| | Breast | Major Cities | 0.007 | 0.002 | 0.013 |
| | | Regional | -0.024 | -0.031 | -0.018 |
| | | Remote | -0.088 | -0.121 | -0.055 |
| | Lung | Major Cities | -0.031 | -0.043 | -0.020 |
| | | Regional | 0.005 | -0.009 | 0.019 |
| | | Remote | 0.060 | 0.003 | 0.116 |
| | Melanoma | Major Cities | -0.122 | -0.144 | -0.100 |
| | | Regional | 0.097 | 0.078 | 0.117 |
| | | Remote | -0.107 | -0.174 | -0.042 |

Table S2: Posterior summary of region specific mean $log(SIR)$ for less common/rare cancers

| Model | Cancer | Region | Mean | 2.5% quantile | 97.5% quantile |
|---|---|---|---|---|---|
| Model 4: Females | Liver | Major Cities | -0.011 | -0.027 | 0.005 |
| | | Regional | -0.183 | -0.202 | -0.164 |
| | | Remote | 0.039 | -0.043 | 0.121 |
| | Oesophageal | Major Cities | -0.050 | -0.063 | -0.037 |
| | | Regional | -0.016 | -0.033 | 0.000 |
| | | Remote | -0.026 | -0.089 | 0.038 |
| Model 5: Males | Brain | Major Cities | -0.010 | -0.016 | -0.003 |
| | | Regional | -0.011 | -0.020 | -0.002 |
| | | Remote | -0.045 | -0.083 | -0.006 |
| | Oesophageal | Major Cities | -0.098 | -0.109 | -0.088 |
| | | Regional | 0.116 | 0.102 | 0.130 |
| | | Remote | 0.242 | 0.191 | 0.294 |
| | Thyroid | Major Cities | 0.013 | -0.002 | 0.027 |
| | | Regional | -0.144 | -0.159 | -0.128 |
| | | Remote | -0.181 | -0.232 | -0.130 |
| Model 6: Females | Brain | Major Cities | -0.004 | -0.010 | 0.002 |
| | | Regional | -0.009 | -0.017 | 0.000 |
| | | Remote | -0.030 | -0.066 | 0.007 |
| | Cervical | Major Cities | -0.043 | -0.056 | -0.031 |
| | | Regional | 0.005 | -0.011 | 0.022 |
| | | Remote | 0.173 | 0.113 | 0.233 |
| | Head & Neck | Major Cities | -0.055 | -0.064 | -0.046 |
| | | Regional | 0.055 | 0.043 | 0.068 |
| | | Remote | 0.188 | 0.137 | 0.239 |
| | Kidney | Major Cities | -0.022 | -0.032 | -0.012 |
| | | Regional | 0.003 | -0.009 | 0.016 |
| | | Remote | -0.047 | -0.097 | 0.003 |
| | Stomach | Major Cities | 0.004 | -0.007 | 0.015 |
| | | Regional | -0.094 | -0.107 | -0.081 |
| | | Remote | -0.139 | -0.189 | -0.091 |
| Model 7: Persons | Brain | Major Cities | -0.004 | -0.009 | 0.001 |
| | | Regional | -0.008 | -0.015 | 0.000 |
| | | Remote | -0.060 | -0.093 | -0.027 |
| | Liver | Major Cities | -0.012 | -0.029 | 0.004 |
| | | Regional | -0.200 | -0.218 | -0.182 |
| | | Remote | 0.093 | 0.005 | 0.181 |
| | Oesophageal | Major Cities | -0.087 | -0.097 | -0.077 |
| | | Regional | 0.110 | 0.097 | 0.123 |
| | | Remote | 0.239 | 0.189 | 0.290 |
| | Stomach | Major Cities | 0.004 | -0.006 | 0.015 |
| | | Regional | -0.072 | -0.084 | -0.060 |
| | | Remote | -0.111 | -0.157 | -0.066 |

Table S3: Posterior summary of region specific mean $log(SIR)$ for smoking related cancers

| Model | Cancer | Region | Mean | 2.5% quantile | 97.5% quantile |
|---|---|---|---|---|---|
| Model 8a: Males | Head & Neck | Major Cities | -0.118 | -0.131 | -0.105 |
| | | Regional | 0.127 | 0.111 | 0.143 |
| | | Remote | 0.403 | 0.342 | 0.464 |
| | Kidney | Major Cities | 0.001 | -0.007 | 0.009 |
| | | Regional | -0.002 | -0.013 | 0.008 |
| | | Remote | -0.080 | -0.124 | -0.036 |
| | Liver | Major Cities | -0.014 | -0.031 | 0.003 |
| | | Regional | -0.210 | -0.230 | -0.191 |
| | | Remote | -0.012 | -0.094 | 0.071 |
| | Lung | Major Cities | -0.062 | -0.076 | -0.047 |
| | | Regional | 0.036 | 0.021 | 0.052 |
| | | Remote | 0.174 | 0.114 | 0.234 |
| | Oesophageal | Major Cities | -0.095 | -0.105 | -0.084 |
| | | Regional | 0.118 | 0.104 | 0.132 |
| | | Remote | 0.244 | 0.192 | 0.296 |
| | Pancreatic | Major Cities | -0.009 | -0.016 | -0.001 |
| | | Regional | -0.015 | -0.026 | -0.005 |
| | | Remote | -0.014 | -0.056 | 0.028 |
| | Stomach | Major Cities | 0.002 | -0.010 | 0.013 |
| | | Regional | -0.068 | -0.081 | -0.055 |
| | | Remote | -0.126 | -0.176 | -0.076 |

Table S3 (Continued) Posterior summary of region specific mean $log(SIR)$ for smoking related cancers

| Model | Cancer | Region | Mean | 2.5% quantile | 97.5% quantile |
|---|---|---|---|---|---|
| Model 8b: Females | Head & Neck | Major Cities | -0.055 | -0.064 | -0.046 |
| | | Regional | 0.055 | 0.042 | 0.067 |
| | | Remote | 0.190 | 0.139 | 0.241 |
| | Kidney | Major Cities | -0.022 | -0.032 | -0.012 |
| | | Regional | 0.004 | -0.009 | 0.016 |
| | | Remote | -0.047 | -0.099 | 0.004 |
| | Liver | Major Cities | -0.009 | -0.025 | 0.007 |
| | | Regional | -0.183 | -0.203 | -0.164 |
| | | Remote | 0.039 | -0.043 | 0.120 |
| | Lung | Major Cities | -0.032 | -0.043 | -0.020 |
| | | Regional | 0.005 | -0.009 | 0.019 |
| | | Remote | 0.060 | 0.003 | 0.116 |
| | Oesophageal | Major Cities | -0.050 | -0.063 | -0.037 |
| | | Regional | -0.016 | -0.033 | 0.000 |
| | | Remote | -0.026 | -0.088 | 0.037 |
| | Pancreatic | Major Cities | -0.009 | -0.015 | -0.003 |
| | | Regional | -0.010 | -0.018 | -0.001 |
| | | Remote | -0.025 | -0.063 | 0.012 |
| | Stomach | Major Cities | 0.005 | -0.005 | 0.016 |
| | | Regional | -0.094 | -0.106 | -0.081 |
| | | Remote | -0.139 | -0.188 | -0.091 |
| Model 8c: Persons | Head & Neck | Major Cities | -0.107 | -0.119 | -0.095 |
| | | Regional | 0.129 | 0.114 | 0.145 |
| | | Remote | 0.437 | 0.378 | 0.496 |
| | Kidney | Major Cities | -0.004 | -0.012 | 0.004 |
| | | Regional | 0.010 | 0.000 | 0.021 |
| | | Remote | -0.076 | -0.121 | -0.030 |
| | Liver | Major Cities | -0.011 | -0.027 | 0.006 |
| | | Regional | -0.194 | -0.212 | -0.176 |
| | | Remote | 0.100 | 0.013 | 0.189 |
| | Lung | Major Cities | -0.050 | -0.063 | -0.037 |
| | | Regional | 0.040 | 0.026 | 0.054 |
| | | Remote | 0.193 | 0.134 | 0.251 |
| | Oesophageal | Major Cities | -0.084 | -0.094 | -0.074 |
| | | Regional | 0.112 | 0.098 | 0.125 |
| | | Remote | 0.240 | 0.190 | 0.292 |
| | Pancreatic | Major Cities | -0.003 | -0.009 | 0.003 |
| | | Regional | -0.012 | -0.020 | -0.003 |
| | | Remote | -0.025 | -0.061 | 0.0112 |
| | Stomach | Major Cities | 0.005 | -0.006 | 0.015 |
| | | Regional | -0.072 | -0.084 | -0.060 |
| | | Remote | -0.111 | -0.157 | -0.066 |

Table S4: Number of SA2s with higher incidence for groups of cancers jointly and individually for most common cancers

| Group | Cancer | No. of SA2s |
|---|---|---|
| Most Common Cancers (for males): Model 1(a) | Lung only | 141 |
| | Melanoma only | 426 |
| | Lung & melanoma | 20 |
| | Bowel only | 265 |
| | Lung & bowel | 76 |
| | Melanoma & bowel | 69 |
| | Lung, melanoma & bowel | 4976 |
| Most Common Cancers (for females): Model 1(b) | Lung only | 17 |
| | Melanoma only | 486 |
| | Bowel only | 206 |
| | Lung & bowel | 4 |
| | Melanoma & bowel | 157 |
| Most Common Cancers (for males): Model 2 | Lung only | 158 |
| | Melanoma only | 211 |
| | Lung & Melanoma | 6 |
| | Bowel only | 259 |
| | Lung & Bowel | 74 |
| | Prostate only | 59 |
| | Lung & Prostate | 50 |
| | Lung, Melanoma & Bowel | 33 |
| | Melanoma & Prostate | 216 |
| | Lung, Melanoma & Prostate | 13 |
| | Bowel & Prostate | 5 |
| | Lung, Bowel & Prostate | 4 |
| | Melanoma, Bowel & Prostate | 23 |
| | Lung, Melanoma, Bowel & Prostate | 12 |
| Most Common Cancers (for females): Model 3 | Lung only | 17 |
| | Melanoma only | 335 |
| | Bowel only | 205 |
| | Lung & Bowel | 4 |
| | Breast only | 68 |
| | Lung & Breast | 156 |
| | Melanoma & Breast | 159 |

Table S5:Number of SA2s with higher incidence for groups of cancers jointly and individually for rare and less common cancers

| Group | Cancer | No. of SA2s |
|---|---|---|
| Rare/ Less common Cancers (females): Model 4 | Liver only | 5 |
| Rare/Less common Cancers (males): Model 5 | Oesophegeal only | 820 |
| | Thyroid only | 261 |
| Less Common/ Rare Cancers (for females): Model 6 | Head & neck only | 378 |
| | Cervical & Head and neck | 79 |
| | Stomach only | 13 |
| Less Common/ Rare Cancers (for persons): Model 7 | Oesophageal only | 17 |
| | Liver only | 765 |
| | Stomach only | 38 |
| | Oesophageal & Liver | 39 |
| | Oesophageal & Stomach | 208 |

Table S6: Number of SA2s with higher incidence for groups of cancers jointly and individually for smoking related cancers

| Group | Cancer | No. of SA2s |
|---|---|---|
| for females | Stomach only | 98 |
| Model 8(b) | Liver & Stomach | 71 |
| | Head and Neck only | 434 |
| | Lung & Head and Neck | 14 |
| | Liver& Head and Neck | 5 |
| | Lung, Liver & Head and Neck | 5 |
| for persons | Lung only | 26 |
| Model 8(c) | Liver only | 11 |
| | Lung & Liver | 2 |
| | Stomach only | 25 |
| | Lung & Stomach | 7 |
| | Liver & Stomach | 130 |
| | Lung, Liver & Stomach | 36 |
| | Lung, Stomach & Pancreatic | 1 |
| | Liver, Stomach & Pancreatic | 12 |
| | Lung, Liver, Stomach & Pancreatic | 15 |
| | Kidney only | 24 |
| | Lung & Kidney | 47 |
| | Stomach & Kidney | 1 |
| | Lung, Stomach & Kidney | 23 |
| | Liver, Stomach & Kidney | 1 |
| | Lung, Liver,Stomach & Kidney | 18 |
| | Lung, Pancreatic,Stomach & Kidney | 2 |
| | Lung,Liver, Pancreatic,Stomach & Kidney | 5 |
| | Oesophageal | 143 |
| | Lung & Oesophageal | 11 |
| | Oesophageal and Kidney | 17 |
| | Lung, Oesophageal and Kidney | 58 |
| | Lung,Head and Neck & Kidney | 2 |
| | Lung, Liver, Kidney & Head and Neck | 15 |
| | Lung, Liver, Pancreatic, Kidney & Head and Neck | 1 |
| | Lung, Liver & Head & Neck | 1 |
| | Lung, Stomach, Kidney & Head and Neck | 9 |
| | Lung, Liver, Stomach, Kidney & Head and Neck | 8 |
| | Lung, Pancreatic, Stomach, Kidney & Head and Neck | 1 |
| | Lung, Liver, Pancreatic, Stomach, Kidney & Head and Neck | 2 |
| | Oesophageal & Head and Neck | 244 |
| | Lung,Oesophageal & Head and Neck | 298 |
| | Liver, Oesophageal & Head and Neck | 5 |
| | Lung, Liver, Oesophageal & Head and Neck | 34 |
| | Kidney, Oesophageal & Head & Neck | 9 |
| | Lung,Kidney, Oesophageal & Head & Neck | 7 |

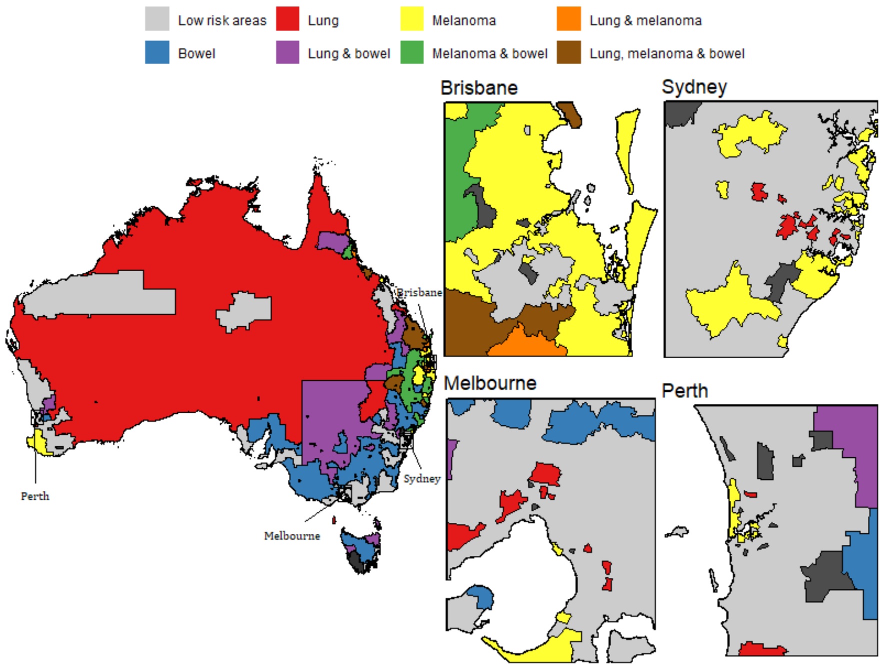


Figure S1: High and low risk areas for multiple cancers (most common cancers for males: Model 1(a))

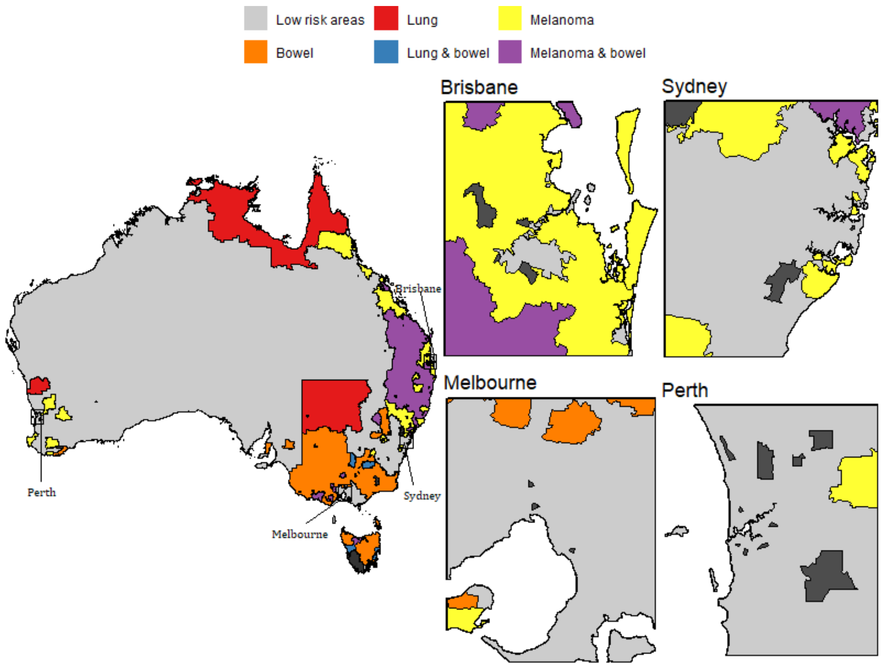


Figure S2:High risk areas for multiple cancers (most common cancers for females: Model 1(b))

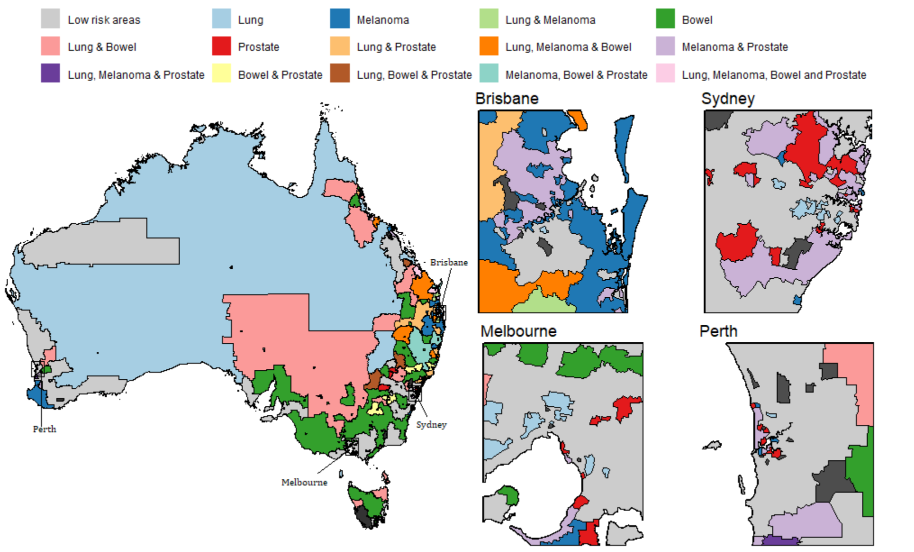


Figure S3: High and low risk areas for multiple cancers (most common cancers for males: Model 2)

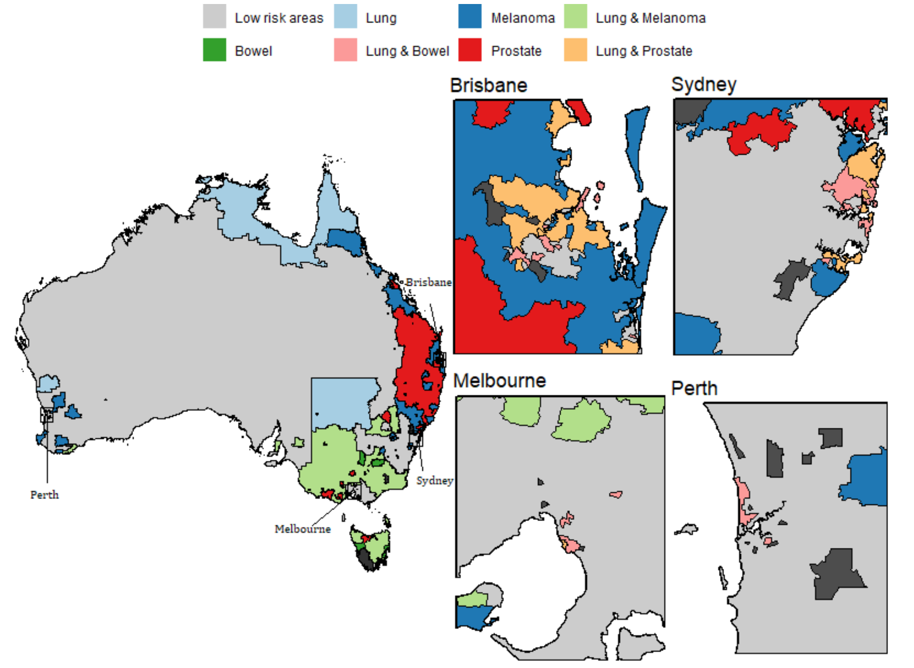


Figure S4: High and low risk areas for multiple cancers (most common cancers for females: Model 3)

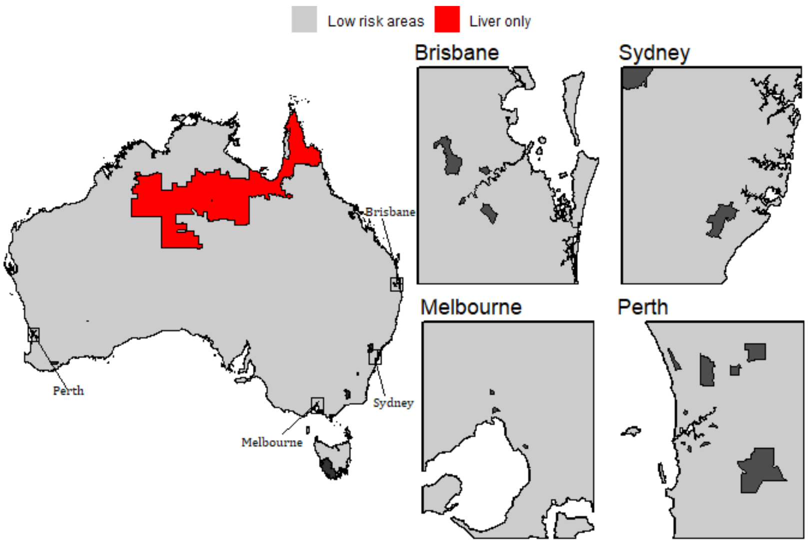


Figure S5: High and low risk areas for multiple cancers (Rare and less common cancers for females: Model 4)

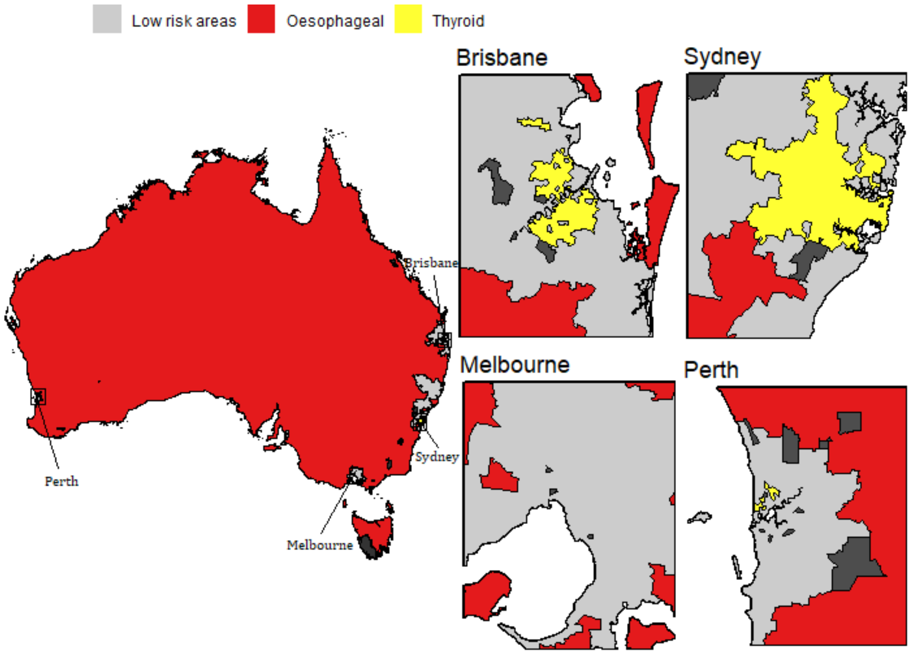


Figure S6: High and low risk areas for multiple cancers (Rare and less common cancers for males: Model 5)

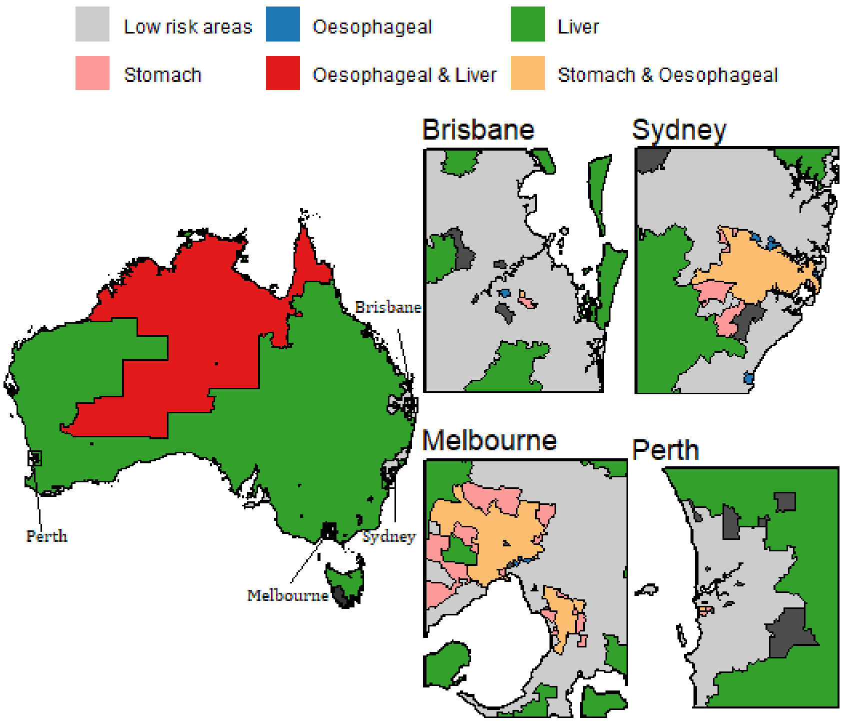


Figure S7: High and low risk areas for multiple cancers (Rare and less common cancers for females: Model 7)

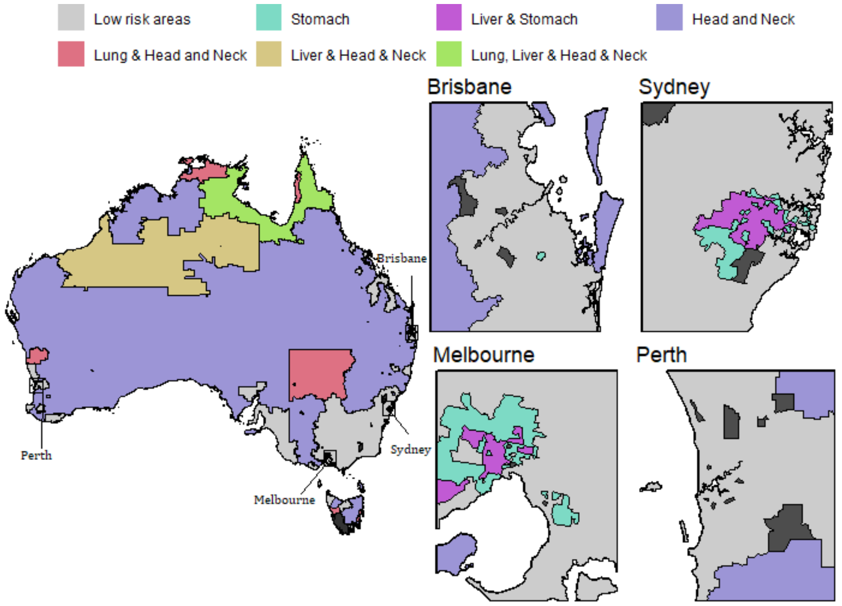


Figure S8: High and low risk areas for multiple cancers (smoking related cancers for females: Model 8b)

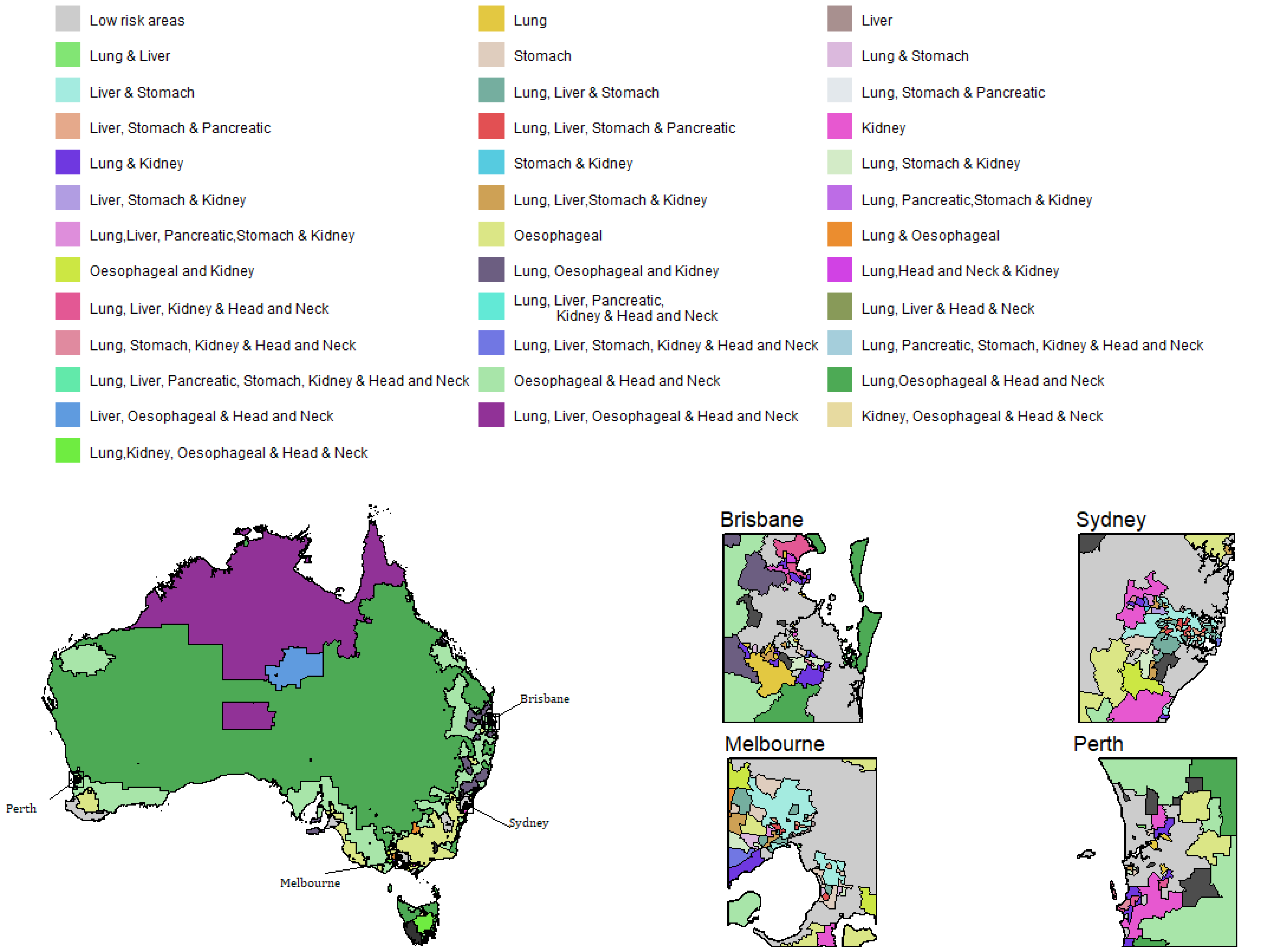


Figure S9: High and low risk areas for multiple cancers (smoking related cancers for persons: Model 8c)

# Multivariate Models for additional groups

## Hormone related cancers

Among the cancer types included in the ACA, hormone related cancers include breast, cervical, ovarian, bowel [1].The proposed model is fitted to this group of cancers for females only (Model 9).

## Overweight and obesity related cancers

Among the cancer types included in the ACA, overweight and obesity related cancers include oesophageal, pancreatic, bowel, breast ovarian and kidney [1]. The proposed model is fitted to two subgroups under this broad group, oesophageal, pancreatic, bowel and kidney for males (Model 10(a)) ; oesophageal, pancreatic, bowel, breast, ovarian and kidney for females (Model 10(b)).

## Results for Model 9 and 10

The posterior means and associated 95% credible intervals for SIRs for models 9 and 10 (a & b) are shown in figures S10-S11. The actual values for posterior $log(SIR)$ can be seen in Table S7. The posterior correlation matrices in major cities, regional and remote Australia for the cancers included in model 9 and 10 (a & b) are shown in Figure S12. The results of Jennrich's test of equality of the correlation matrices for the remoteness regions are shown in Table S8. The number of areas with high risk of cancer incidence for both hormone related and obesity & overweight related cancers are shown in Table S9. The spatial maps visualising the high and low risk areas are presented in figures S13 - S15.

Table S7: Posterior summary of region specific mean $log(SIR)$ for Hormone related (Model 9) and
overweight & obesity related cancers (Model 10)

| Model | Cancer | Region | Mean | 2.5% quantile | 97.5% quantile |
|---|---|---|---|---|---|
| Model 10: Females | Bowel | Major Cities | -0.029 | -0.034 | -0.023 |
| | | Regional | 0.044 | 0.035 | 0.052 |
| | | Remote | -0.025 | -0.062 | 0.012 |
| | Breast | Major Cities | 0.007 | 0.002 | 0.012 |
| | | Regional | -0.024 | -0.031 | -0.018 |
| | | Remote | -0.088 | -0.122 | -0.055 |
| | Cervical | Major Cities | -0.044 | -0.056 | -0.031 |
| | | Regional | 0.004 | -0.012 | 0.021 |
| | | Remote | 0.173 | 0.114 | 0.233 |
| | Ovarian | Major Cities | -0.003 | -0.009 | 0.003 |
| | | Regional | -0.024 | -0.032 | -0.016 |
| | | Remote | -0.049 | -0.085 | -0.013 |
| Model 10(a): Males | Bowel | Major Cities | -0.027 | -0.033 | -0.021 |
| | | Regional | 0.044 | 0.036 | 0.053 |
| | | Remote | -0.026 | -0.063 | 0.012 |
| | Kidney | Major Cities | -0.031 | -0.043 | -0.020 |
| | | Regional | 0.005 | -0.009 | 0.019 |
| | | Remote | 0.060 | 0.004 | 0.118 |
| | Oesophageal | Major Cities | -0.119 | -0.141 | -0.097 |
| | | Regional | 0.098 | 0.078 | 0.117 |
| | | Remote | -0.107 | -0.173 | -0.0414 |
| | Pancreatic | Major Cities | -0.119 | -0.141 | -0.097 |
| | | Regional | 0.098 | 0.078 | 0.117 |
| | | Remote | -0.107 | -0.173 | -0.0414 |
| Model 10(b): Females | Bowel | Major Cities | -0.028 | -0.036 | -0.019 |
| | | Regional | 0.045 | 0.032 | 0.058 |
| | | Remote | -0.026 | -0.104 | 0.051 |
| | Breast | Major Cities | 0.008 | 0.000 | 0.016 |
| | | Regional | -0.024 | -0.036 | -0.013 |
| | | Remote | -0.088 | -0.161 | -0.013 |
| | Pancreatic | Major Cities | -0.009 | -0.018 | 0.001 |
| | | Regional | -0.011 | -0.024 | 0.002 |
| | | Remote | -0.025 | -0.104 | 0.053 |
| | Kidney | Major Cities | -0.022 | -0.034 | -0.009 |
| | | Regional | 0.005 | -0.013 | 0.022 |
| | | Remote | -0.048 | -0.137 | 0.040 |
| | Oesophageal | Major Cities | -0.049 | -0.065 | -0.033 |
| | | Regional | -0.014 | -0.036 | 0.007 |
| | | Remote | -0.026 | -0.126 | 0.078 |
| | Ovarian | Major Cities | -0.002 | -0.011 | 0.007 |
| | | Regional | -0.024 | -0.036 | -0.011 |
| | | Remote | -0.048 | -0.128 | 0.030 |

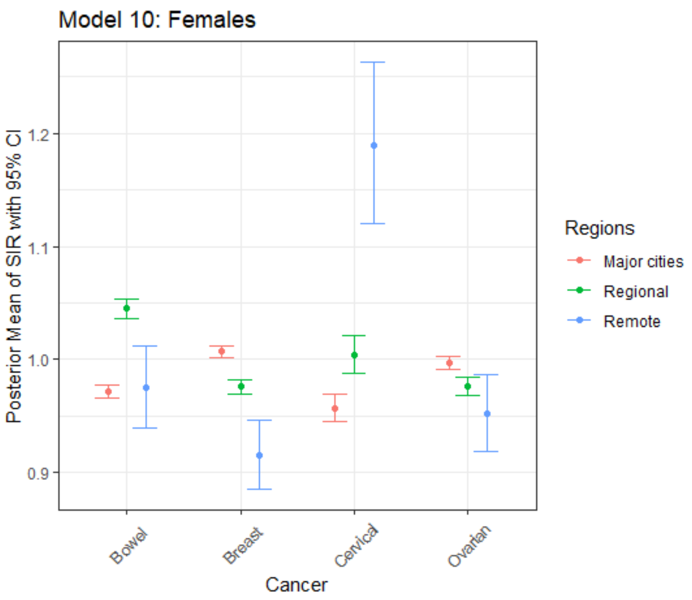


Figure S10: Posterior means with 95% credible intervals of SIR for the hormone related cancers over remoteness regions, Australia

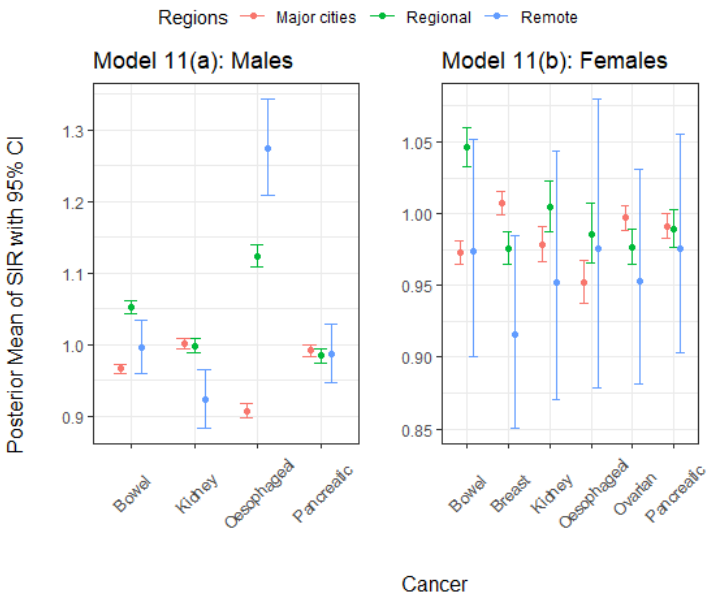


Figure S11: Posterior means and 95% credible intervals of SIR for the overweight and obesity related cancers over remoteness regions, Australia

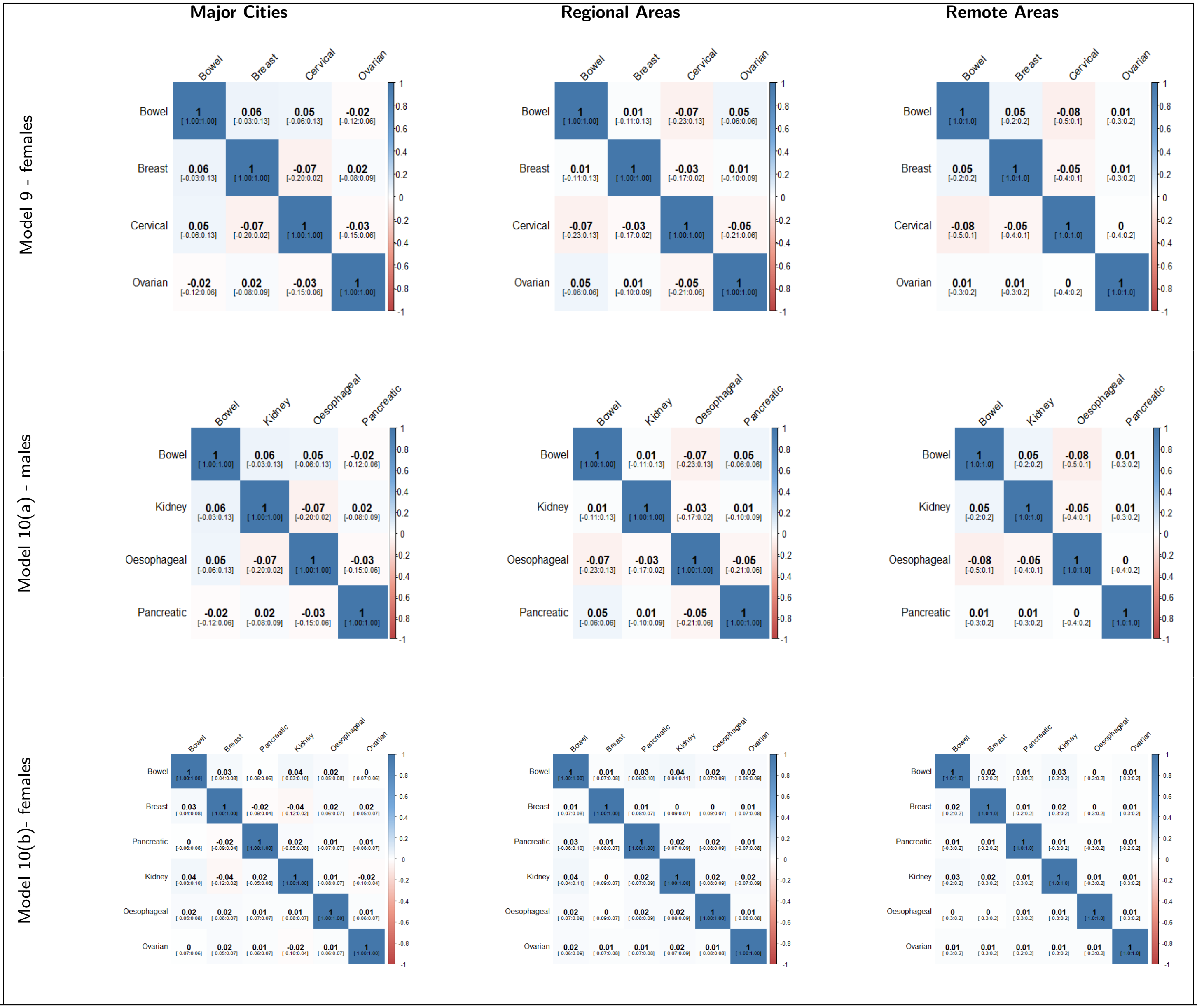


Figure S12: Posterior Correlation matrices with 95% credible intervals for hormone related cancers (Model 9) and overweight & obesity related cancers (Model 10 a & b) by region

Table S8: Results of Jennrich's Test of differences in correlation matrices applied to hormone related and obesity & overweight related cancers in different remoteness regions

| Group | Model | Test statistic [a] | Critical_value | P_value |
|---|---|---|---|---|
| Hormone related cancers | Model 10: Females | 12.87 | 21.03 | 0.38 |
| Overweight and obesity related cancers | Model 11(a): Males | 23.96 | 21.26 | 0.02 |
| | Model 11(b): Females | 3.42 | 43.77 | 0.99 |

[a] Null Hypothesis: Equality of correlation matrices in major cities, regional and remote areas for each group of cancers

Table S9: Number of SA2s with higher incidence for groups of cancers jointly and individually, models 9 and 10

| Model | Cancer | No. of SA2s |
|---|---|---|
| Model 9: females | Low risk areas | 1608 |
| | Bowel only | 362 |
| | Breast only | 100 |
| | Cervical only | 78 |
| Model 10(a): males | Low risk areas | 1287 |
| | Bowel only | 279 |
| | Kidney only | 1 |
| | Oesophageal only | 395 |
| | Bowel & Oesophageal | 436 |
| Model 10(b):females | Low risk areas | 1673 |
| | Bowel only | 260 |
| | Breast only | 177 |
| | Pancreatic only | 10 |
| | Bowel& Pancreatic | 9 |
| | Kidney only | 1 |
| | Bowel & Kidney | 1 |
| | Ovarian | 14 |

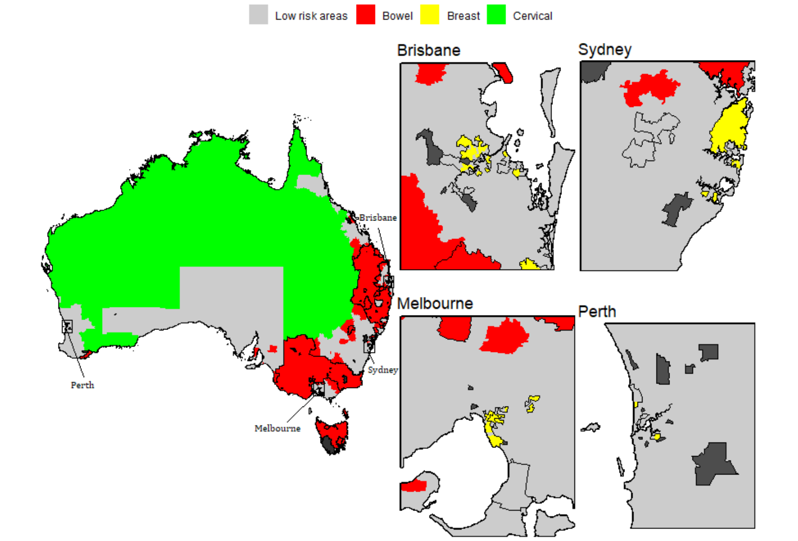


Figure S13: High and low risk areas for individuals and multiple cancers (hormone related cancers: Model 9)

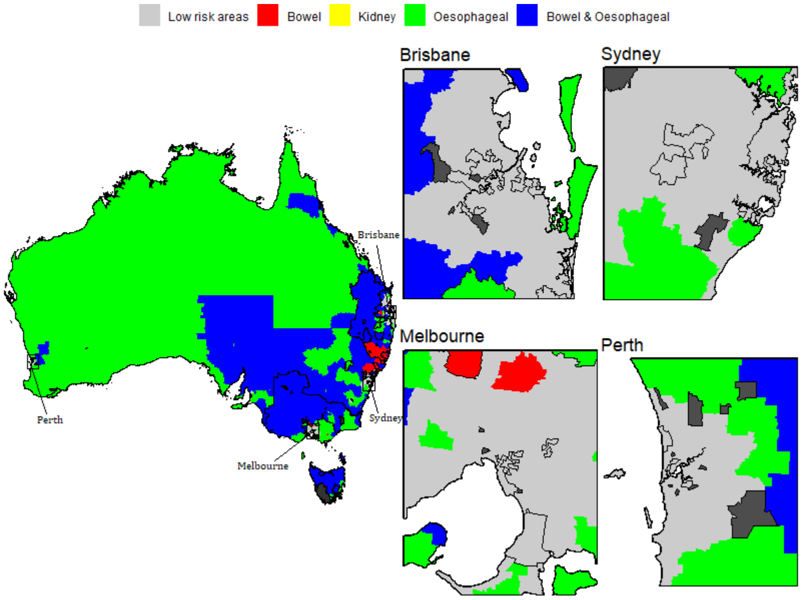


Figure S14: High and low risk areas for individuals and multiple cancers (obesity and overweight related cancers for males: Model 11(a))

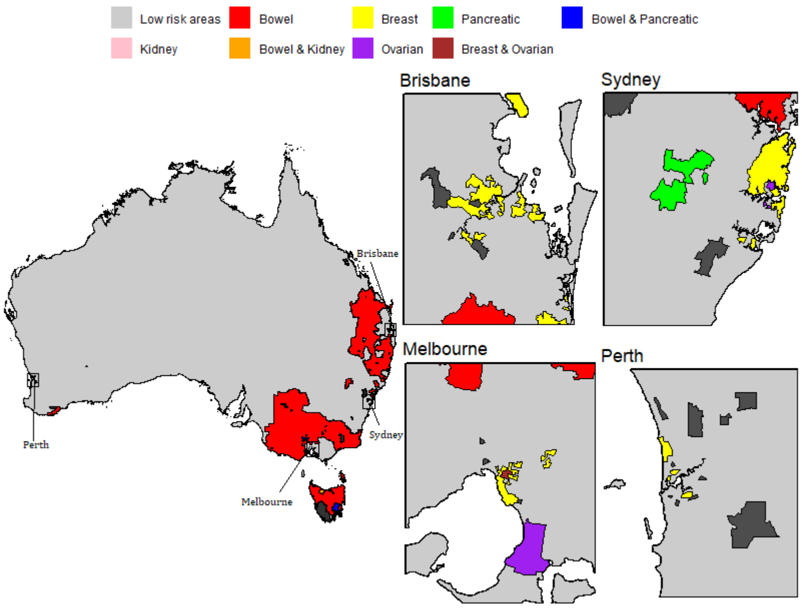


Figure S15: High and low risk areas for individuals and multiple cancers (obesity and overweight related cancers for males: Model 11(b))

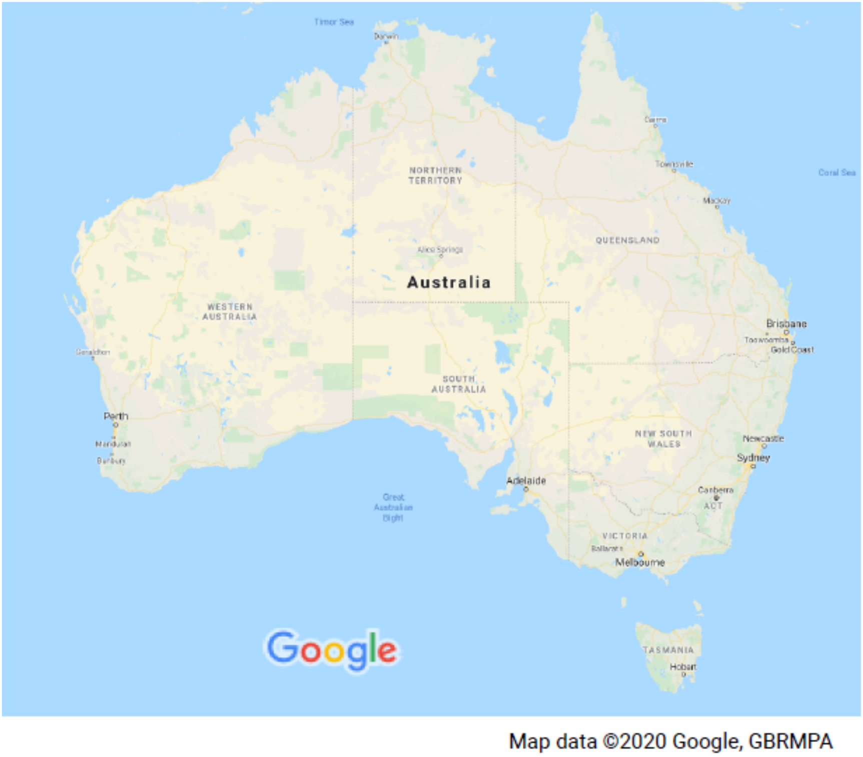


Figure S16: Map of Australia showing states and capitals

**Author details**
[1]ARC Centre of Excellence in Mathematical and Statistical Frontiers, School of Mathematical Sciences, Science and Engineering Faculty, Queensland University of Technology, QLD 4001 Brisbane, Australia. [2]Institute of Health and Biomedical Innovation, Queensland University of Technology, QLD 4001 Brisbane, Australia. [3]Cancer Council Queensland, 553 Gregory Terrace, Fortitude Valley QLD 4006 Brisbane, Australia.

**References**
1. Whiteman DC, Webb PM, Green AC, Neale RE, Fritschi L, Bain CJ, et al. Cancers in Australia in 2010 attributable to modifiable factors: introduction and overview. Australian and New Zealand journal of public health. 2015;39(5):403–407. Available from: https://www.ncbi.nlm.nih.gov/pmc/articles/PMC4606764/.
